# *“There’s no representation”:* a qualitative study of attitudes and motivations towards genomic research participation among Australian South Asians

**DOI:** 10.1038/s41431-026-02129-3

**Published:** 2026-05-21

**Authors:** Vaishnavi Nathan, Heena Akbar, Aideen McInerney-Leo, Deborah Gilroy, Anjali Henders, Reema Naresh, Nahid Choudhury, Maleeha Waqar, Sonia Shah, Tatiane Yanes

**Affiliations:** 1 Frazer Institute, The University of Queensland, Dermatology Research Centre, Brisbane, QLD Australia; 2 School of Public Health, Faculty of Health, Medicine and Behavioural Science, The University of Queensland, Brisbane, QLD Australia; 3 Institute for Molecular Biosciences, University of Queensland, Brisbane, QLD Australia; 4 Brisbane Northside Fiji Senior Citizens, Brisbane, QLD Australia; 5 Metro North Mental Health, School of Health Sciences, Department of Nursing and Allied Health, Swinburne University of Technology, Melbourne, VIC Australia; 6 School of Nursing, Queensland University of Technology, Kelvin Grove, QLD Australia; 7 School of Biomedical Sciences, The University of Queensland, Brisbane, QLD Australia; 8 The University of Queensland Faculty of Health, Medicine, and Behavioural Sciences, Cardiology Department, Royal Brisbane and Women’s Hospital, Brisbane, QLD Australia; 9 Department of Cardiology, Royal Brisbane and Women’s Hospital, Brisbane, QLD Australia; 10 Sunshine Coast University Hospital, Birtinya, QLD Australia; 11 Genetic Health Queensland, Royal Brisbane and Women’s Hospital, Herston, QLD Australia; 12 School of Biomedical Sciences, Faculty of Health, Queensland University of Technology, Brisbane, QLD Australia; 13 QIMR Berghofer, Brisbane, QLD Australia; 14 General Practice Clinical Unit, Medical School, The University of Queensland, Brisbane, QLD Australia

**Keywords:** Genetics research, Psychology

## Abstract

People of South Asian ancestry represent approximately 25% of the world’s population, yet constitute less than 2% of global genomic databases, limiting our ability to provide equitable genomic healthcare for this population. The urgent need to improve representation of diverse populations in genomic research is widely recognised as an area of priority among the genetics community. Community engagement is a key first step to informing tailored recruitment strategies and genomic research participation. This study aimed to understand prior experience with, and attitudes towards, genomic research within the context of cardiovascular disease risk among people of South Asian ancestry residing in Queensland, Australia. Semi-structured focus groups were conducted between April and August 2023 (*n* = 60 individuals), which were recorded, transcribed verbatim and analysed using inductive and deductive approaches. Three thematic categories were developed: ‘*Engagement with Genomic Research*’, ‘*Cultural Connections*’ and ‘*Trust and Relationship Building*’. While participants expressed positive views toward genomic research, only a few individuals had previously participated, primarily due to a lack of awareness and engagement from researchers in genomic studies. Fear of stigma and discrimination was a significant determinant towards genomic research engagement, which was multi-faceted and rooted in both community-level concerns and lived experiences of racial discrimination in Australia. Conversely, community partnership and establishing trustworthiness were critical facilitators for enhancing participation in genomic research. These findings will have important implications for designing culturally responsive community engagement strategies and will inform the development of recruitment protocols tailored to South Asian communities in Australia.

## Introduction

Genomic research investigates how variations in the human genome influence phenotypic variation between individuals, including differences in disease susceptibility. While the benefits of genomic research and subsequent clinical implications are well recognised, there are limitations to this technology for individuals of non-European ancestry [1, 2]. Interpretation of genomic data relies on established databases that are predominantly comprised of data from European populations [1, 2]. Differences in variant frequencies and effect sizes, as well as missed variants in underrepresented populations, can result in less equitable testing for those of non-European ancestry. For example, compared to people of European ancestry, those of non-European ancestry are more likely to receive non-clinically significant results or have variants of unknown significance reported [2]. Hence, the urgent need to improve representation of diverse populations in genomic research has been widely recognised as an area of priority by the genetics community [3–5].

Several frameworks have been developed to engage diverse communities in genomic research [2, 6, 7]. These frameworks have been frequently adapted from community-based participatory research (CBPR), a collaborative approach that incorporates community partnership to achieve equitable outcomes for research and healthcare [8]. Principles of CBPR emphasise respect for local context that is geared towards building strengths and resources within the community, long-term partnerships, and equity between researchers and the community across all phases of the research [8, 9]. Within the genomic setting, several studies have successfully applied CBPR principles to create meaningful community partnerships and inform clinical practice and research projects, with strategies including establishing community advisory groups (CAG) and working with community partners to inform study design, recruitment, data analysis, and resource development [10–13]. Overall, use of CBPR increases research recruitment and retention, reduces access disparities, and improves uptake of healthcare treatments [14, 15].

People of South Asian ancestry (ancestry from India, Pakistan, Bangladesh, Sri Lanka, Nepal, and Bhutan) represent an ethnolinguistically diverse population, marked by unique genomic characteristics [16, 17]. However, this population is severely underrepresented in genome research, consisting of less than 2% of published genome-wide association studies, while representing 25% of the global population [1]. Furthermore, South Asians have distinct risk profiles for cardiovascular disease (CVD), increased disease risk from a younger age, and are more likely to present with adverse events such as myocardial infarction and stroke [18, 19]. Recognising these healthcare needs, international initiatives such as the Genes & Health Study (UK) [20], GenomeIndia (India) [21], and the Mediators of Atherosclerosis in South Asians Living in America (MASALA; USA) [22] were established. The Genes & Health Study has successfully recruited nearly 72,000 participants of Bangladeshi and Pakistani heritage, contributing to studies across community priority areas such as diabetes, mental health, heart disease and cancer [20].

South Asians make up the largest non-European overseas-born population in Australia [23]. The South Asian Genes and Health in Australia Study (SAGHA), established in 2023, aims to improve participation of Australian South Asians in genomic and CVD research. Our goal is to better our understanding of the genetic and non-genetic contributions to CVD for South Asians in Australia, thereby improving health outcomes and health equity. The present study reports findings from focus groups that aimed to understand social attitudes and the context of genomic research among South Asians living in Queensland, Australia. Findings will inform the development of a community engagement framework for genomic research with South Asian communities. We explored the following research questions:

1. What are the attitudes, perceptions and views of Australian South Asians residing in Queensland regarding genomic research participation?
2. What are the barriers and facilitators of Australian South Asian participation in genomic research?

## Methods

### Study design

CBPR principles and frameworks for promoting diversity in genomic research [6, 8] were embedded in the SAGHA study design from the onset [8]. Specifically, the researcher-community relationship and meaningful engagement were a priority area for SAGHA design. The inclusion of South Asian researchers, South Asian-led funding, and the establishment of a CAG from the grant writing stage, with specific CAG funding allocated post-award, were key drivers in ensuring community voices were embedded in the study. The CAG provided input on the study design, participant-facing documents, data collection and analysis, and manuscript write-up, with members invited for authorship.

### Participant eligibility

Eligibility participants were aged >18 years, self-identified as South Asian ancestry (i.e. from India, Pakistan, Bangladesh, and Sri Lanka) and residing in Queensland, Australia. Ancestry was defined based on the country or geographical location from which participants were biologically descended [24]. Although countries such as Nepal and Bhutan are geographically located within South Asia, their genetic ancestry includes significant East Asian admixture and therefore were not recruited into this study. Ethnicity is recognised as a concept distinct from ancestry, defined as a social construct encompassing shared cultural characteristics such as language, practices, and beliefs [24]. Ethnicity data was collected to inform participant recruitment, focus group enrolment, data analysis, and assist with future SAGHA initiatives. Participation was not restricted based on prior experiences with health or genomic research. Individuals were eligible regardless of English proficiency, with funding allocated to provide interpreter support where needed.

### Recruitment

An online expression of interest (EOI) form was distributed through researchers, CAG members and community leaders, which outlined the study purpose, research team and setting, and collected basic demographic information (i.e. ancestry, interpreter support needs, and gender preferences for focus groups). Self-reported ethnicity was captured through open-text boxes. In response to community leaders’ guidance, same gender focus groups were facilitated for open dialogue. Purposive sampling ensured representation across ancestry, gender, age, and South Asian ethnicities. Recruitment was further facilitated through community events, connecting with community leaders, and advertising via media/social media, including The University of Queensland newsletter, LinkedIn and community Facebook and WhatsApp groups. A snowballing approach was also applied, with some participants acting as community liaisons. All individuals provided informed consent prior to attending focus groups, which were conducted between April and August 2023.

### Focus groups

Focus groups were conducted both virtually through video conferencing (Zoom) and in-person at community venues and places of worship. Where a mutually agreed time for group participation could not be arranged, one-on-one interviews were offered and conducted by author VN. While individual interviews lacked group interactions, they were considered appropriate for inclusivity and to ensure all perspectives were captured. Community-specific focus groups were also held to support shared dialogue and perspectives. All focus groups were conducted by authors VN and SS, with two Indo-Fijian focus groups also co-conducted by authors RN and HA.

A PowerPoint presentation (Supplementary Materials 1) was used to guide focus group discussions. Presentation slides evolved over time to address educational gaps that had arisen in previous focus groups. Each session began with an overview of the study aims, participant introductions and a brief discussion of prior research participation. Participants were then shown slides covering genomic in healthcare, the impact of genetic information on CVD disease risk, and the limitations of current genomic testing for individuals of South Asian ancestry. Group discussions were guided by open-ended questions to explore community attitudes toward and engagement with genomic research. Authors VN and SS took observational notes during the sessions, documenting reflections, non-verbal cues, and key participant comments. Whiteboards were used to facilitate discussions, with photographs taken of the content at the conclusion. All participants received a $30 GiftPay E-Gift card in appreciation of their time.

Audio recordings were transcribed verbatim and de-identified with pseudonyms by author VN. Cultural terms and phrases used by participants in their native languages were noted and translated (e.g mandhir (Hindu temple), gurdwara (place of worship in the Sikh faith), Poila Boishakh (Bengali New Year), and pol sambol (Sri Lankan coconut dish)).

### Data analysis

Data was analysed using inductive content analysis [25], informed by the study’s research questions and existing literature on community engagement and genomic research. Initial coding domains were guided by these concepts, while remaining open to new categories emerging directly from the data. This approach allowed for the emergence of unanticipated insights directly from the data, ensuring that participants’ voices and lived experiences shaped the findings. All transcripts were independently reviewed and coded by authors VN, TY, and HA to identify initial codes. The authors then met on several occasions to review initial codes and group these into preliminary thematic categories. Subthemes were developed, merged or separated based on the data and team consensus.

Genetic ancestry and sociodemographic data were used to inform data analysis, including contextualisation of participants experiences and identify divergent perspectives that may shape engagement with genomic research. Field notes taken during focus groups were also used to supplement analysis, particularly for interpreting non-verbal cues and cultural references. NVivo 11 software was used to organise data into codes and thematic categories [26]. The analysis was iterative, with thematic categories and sub-themes refined during the manuscript preparation process. Results were reviewed by all authors and members of the CAG to ensure accurate interpretations within the cultural context of the study participants.

### Team positionality and roles

The research team comprised investigators from diverse professional and cultural backgrounds, including genetic counsellors (authors VN, AML and TY), genomic researchers (authors SS and AH), public health researchers (HA), research nurse (DG), community engagement specialist (RN) and CAG members (NC and MW). Most investigators were of South Asian ancestry (authors VN, HA, AH, RN, NC, MW, and SS), which supported culturally informed approaches across all stages of the project and enhanced understanding of contextual factors discussed in the study. Additionally, the shared cultural background between the research team and participants likely facilitated rapport and openness during focus groups. However, the shared cultural perspectives may also have shaped how data were elicited and interpreted by the research team. For example, influencing the perceived value and acceptability of genomic research. To address this issue, the team engaged in ongoing reflexive discussions throughout the research process, involved multiple researchers in coding and result interpretation, and sought to represent a diversity of perspectives within the data.

## Results

Of 78 EOI received, 60 (77%) individuals participated in the study (*n* = 9 focus groups, and *n* = 1 one-on-one interview). Among non-participants, one person was deemed ineligible due to residing interstate, two withdrew from the study after consent, and 13 were unavailable or did not reply to study invitations. No participant indicated a need for an interpreter in the EOI. However, two individuals who attended focus groups with a relative required an interpreter. As the team was unable to accommodate this request on the day, these individuals were unable to participate in the discussion. Thus, all focus groups were conducted in English. Duration of focus groups ranged from 1.5 to 2 h, including a short break.

**Fig. 1:**
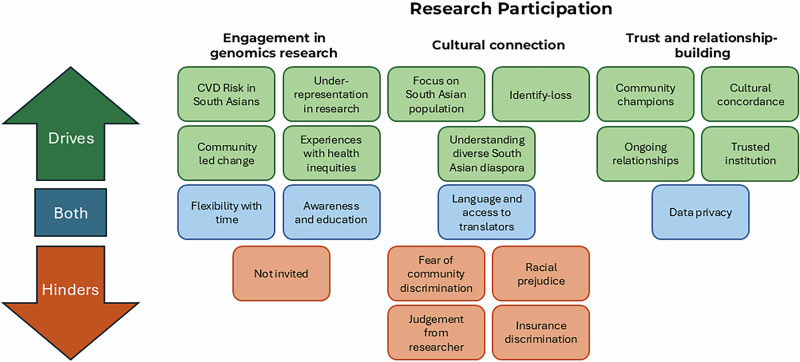
Summary of study thematic categories and subthemes regarding factors that contribute to genomics research participation within the South Asian population in Australia.

All four ancestral groups were represented in focus groups. Most participants were of Indian ancestry (*n* = 43; 72%), female (*n* = 39; 65%) and between the ages of 30–49 (*n* = 35; 58%) (Table 1). Participants self-reported ethnicity from ten unique ethnolinguistic groups, with some identifying from >1 group. Most participants (83%) identified as being 1st generation migrants, either migrating on a skilled work or student visa. Only four participants (7%) were 2nd-generation migrants, with the remaining six participants not reporting their professional or residency status. Three thematic categories were developed, which captured participants’ experiences and attitudes towards genomic and health research: (i) engagement in genomic research, (ii) negotiating cultural identity, and (iii) trust and relationship-building. Collective thematic categories and sub-themes were mapped to describe the factors that hinder and drive engagement of Australian South Asians in genomic research (Fig. 1).

**Table 1:** Characteristics of Participant Demographics.

Demographic Characteristics	*n*	%
*Biological sex*		
Female	39	65
Male	21	35
*Ancestry*		
India	43	72
Bangladesh	7	12
Sri Lanka	5	8
Pakistan	5	8
*Self-reported ethnicity*^a^		
Bengali	3	5
Gujarati	1	2
Marathi	1	2
Indo-Fijian	19	32
Kannadigas	1	2
Malayali	2	3
Punjabi	12	20
Sinhalese	2	3
Tamil	9	15
Telugu	2	3
Not reported^b^	11	18
*Age*		
18–29	7	12
30–39	23	38
40–49	12	20
50–59	6	10
60+	12	20

^a^values sum more than 100% as individuals reported having more than one ethnicity;

^b^not reported at time of EOI or during focus group introductions

### Engagement in genomic research as a negotiated process shaped by awareness, relevance and structural vulnerability

‘Engagement in Genomic research’ describes participants’ prior experiences, attitudes towards, and suggested strategies to support genomic research participation (Table 2). Nearly all participants had little to no experience in health or genomic research, and for most, SAGHA was their first research interaction. Most individuals attributed their limited participation in research to a lack of awareness or invited opportunities (Table 2A). Among the minority of individuals who had been previously invited to participate in health research, all but one had declined due to lack of personal relevance or benefit to them, hesitation to engage in invasive studies, and/or fear of possible negative outcomes for visa applications (Table 2B). Only one person reported prior participation in a genomic study, which was motivated by the desire to obtain information about their health.

**Table 2:** Representative quotes for the theme Engagement in Genomics research*.

Sub-theme	Quote
**Experience with research**	
A. Never invited	*‘I never got any invitation to participate in that kind of study.’* (Usman*, Male, 40-49 years) *‘I guess for me its just that I haven’t come across it…its just never crossed my path’* (Madhumita*, Female, 40–49 years)
B. Previously declined health research participation	‘*I wouldn’t do humanised trials and stuff like that, so just something where it’s not going to cause me any harm*.’ (Vanaja*, Female, 30–39 years). ‘*The other reason* [I did not participate in a research study] *was I was a little scared because we were interested in applying for permanent residency at that time and I was a little scared…of these studies that would hinder our application…’* (Aamira*, Female, 30–39 years).
**Attitudes towards genomic research**	
C. CVD risk in South Asian communities	‘This [research] interests me a lot because heart disease is actually really prevalent in my family….‘I’m trying to learn more about and find more information about heart disease in Indians.’ (Kara*, Female, 30–39 years) *I think that’s the greatest motivator, if you know what impacts you personally, you’re bound to participate… Every day, millions of people die in India due to heart attacks, so my involvement … this is one of the biggest factors* ‘ (Tejinder*, Male, 30–39 years)
D. Strong sense of community	‘…I think we all do believe that this [genomic research] can help someone down the line, maybe, so even our family or…anybody from South Asia, but I suppose other countries; if my involvement now can help one, two, three, four people, even after 10 years, it’s worth it.’ (Irfan*, Male, 30–39 years). *‘I think that’s such a unique part of being South Asian is that it appeals to your sense of community and wanting to do something for the greater good.’* (Dharsha*, Female, 30–39 years)
E. Lack of representation driving interest in genomic research	‘It’s really shocking that, given such a massive part of the population…there’s no representation in the studies and in the data on this very important cause. We definitely need to change that. (Dharsha*, Female, 30–39 years) *‘I think things are not going to get better if we do not participate, get involved, and help the ones who are trying to help us*.’ (Aamira*, Female, 30–39 years).
F. Impact of being underrepresented in healthcare	*‘I could totally relate to what you said about the health charts for babies, I had clinic nurses convincing me that I was starving my babies, and I remember going to my GP and just going, I don’t know what else to do, and she was like, he’s making his own chart…So I stopped going to the health clinics after that.’* (Trisha*, Female, 30–39 years). ‘I myself am a cancer survivor… the tool that you mentioned, the prediction tool, that even for cancer, was specifically developed for [European] women. And they use it on us?’ (Huda*, Female, 40–49 years).
**Strategies to facilitate engagement with research**	
G. Community awareness	‘We would be more willing to participate, just because we know the value of it [genomic research] more. But it's like we need the awareness of it more as well’ (Yamuna*, Female, 30–39 years). *‘I didn’t know that the current set of standards is based on Western data, and data from the South Asian part is not even included while producing this calculator…We are not aware of this discrimination. So I think there should be some sort of awareness program. So if people are aware, like people from the South Asian part are aware that their data is not represented in the health system, or calculating the risk factors of different diseases, then they will be more interested, like…then we will benefit from that research and or participation…* (Hamza*, Male, 30–39 years)
H. Education	*‘Education. That’s the first part. Knowing what it is. No one is going to educate us like you have explained everything to us with the data, about the* [research project]. *That’s the main hurdle. So for me, it’s all about the education- someone is going to explain to me generally about this* [research]. (Kuldeep*, Male, 30–39 years) *The other thing is education. People don’t have the knowledge about what this research could benefit us. So if we have the opportunity, if we could have the research done, I think, it’s good for our coming generations. I mean, it will benefit everyone. ‘* (Noor*, Female, 30–39 years)
I. Flexible time commitment	‘*I think for me, it would be good if you could do everything online. Because I haven’t got any free time to actually go somewhere, because getting out of the house and then the travel time and then you’re getting back… all that counts*.’ (Madhumita*, Female, 40–49 years) *‘I think the restriction would be in terms of flexibility because I work, so it was a bit hard for me to sort of come in at 5:30 pm to do this*’ (Selvi*, Female, 50–59 years) ‘*Time is the biggest factor, how long maybe, how much time, how frequently…’* (Vanaja*, Female, 30–39 years)

^*^pseudonyms provided in quotes

After being provided with an explanation about genomic research and the SAGHA project, nearly all participants expressed strong interest in being part of future genomic studies. This interest was driven by a desire to improve personal healthcare and strong personal, familial, and community experiences with CVD risk (Table 2C). Many participants frequently noted altruistic feelings towards their families and the broader South Asian community, which was recognised as having a high prevalence of CVD risk (Table 2D, E). Most participants were shocked to learn about the lack of South Asian representation in genomic research, prompting some to share personal stories of how underrepresentation had affected their healthcare in other settings (Table 2F). This discussion fostered a sense of responsibility among many to address the underrepresentation of South Asians in genomic research, who also highlighted the importance of the community-led change (Table 2, quotes 2B, C). Similarly, nearly all individuals felt they were representing their community by participating in a study about, and for, South Asians. Upon study participation, several individuals volunteered to reach out to their community to increase representation of their ethnic group (Table 2, quotes 2 G).

Lack of community awareness was highlighted as a key factor hindering engagement in genomic research, with many noting the need to improve community education about the issue (Table 2, quotes 2 G). Participants frequently noted the value of the education received as part of the focus groups, which consequently drove their interest in participating in the SAGHA study. Thus, highlighting an important role for researchers in community engagement and education (Table 3H). When discussing information needed to enrol in genomic research, participants stressed the importance of clearly outlining study expectations, including time commitments. They also valued flexibility and convenient options for those with work and family responsibilities, such as a ‘one-stop-shop’ approach (Table 2I).

**Table 3:** Representative quotes for theme Negotiating cultural identity*.

Sub-theme	Quote
**Cultural heritage and identity**	
A. Cultural connection	*‘I think what drew me to this one was the emphasis on being South Asian because there are so many things that are skewed towards the Western and European population…Otherwise, it sort of feels like my data will get lost amongst the European Western data, so there’s really no point in a really broad general study.’* (Heneesha*, Female, 18-29 years). ‘*I never really came across something that moved me enough to participate until this one* [study] (Dharsha*, Female, 30–39 years)’
B. Unique cultural identities	‘*South Asians, we’re different…, we are so different in our mental health, physical health, we are very different. You can’t lump us all in one group.’* (Sonu*, Female, 60–69 years)
C. Communication	*‘But then there would be many people who are just not here* [focus group] *because they couldn’t explain it in their language’* (Asha*, Female, 30–39 years) *‘No, language is a very big barrier.’* (Ajeet*, Male, 30–39 years). *‘If you see our parents, they don’t understand English that much, so we could help our parents. But if the presentation is done in Hindi or Punjabi so they could understand, that will work, I think. I think having [questionnaires] in English would be fine, but just the presentation, so they just understand what exactly this is about*. ‘ (Manpreet*, Female, 30–39 years)
D. No concerns with the biological sample	*‘I don’t have any reservations. I don’t mind taking blood.’* (Asanka*, Female, 40–49 years)
E. Loss of identify	‘*I believe, like people from Fiji…we sort of lose a lot of identity, because when we come to Australia, we are classified as Indians. But if you look at us very closely, we are sort of more Pacific people…even though we have an Indian heritage. But what happens is our food and everything is quite different from people in India, like the way we live, what we eat, and our lifestyle is quite different. So by us participating in something like this, we’ll probably help our diaspora in the future…’* (Roshan*, Male, 50–59 years). *‘…That sort of history with the migration and the changes and that* [family history information] *is really hard to track. …I’m not sure how much medical knowledge is there and whether it gets passed down and how it gets lost when people move here…’* (Kara*, Female, 30–39 years) *‘Anything particular with my homeland, any group, any information, that’s one of the triggers* [to participating], *because I wanted to go back to my country, my family doesn’t want to go back. So, there’s always some interest to find something, any collaborative approach or any sort of function, because I stay here* [in Australia]*…* (Zaheer*, Male, 30–39 years)
**Fear of stigma and discrimination**	
F. South Asian community	‘Because culturally we don’t want to say anything negative… and so they don’t want the word to get around, he might not be able to get a good girl, he might have trouble getting a job, whatever it is. That fear…’ (Sonu*, Female, 60–69 years) ‘I think the discussions that you’re having, maybe the community events may not be the right place, because people would sort of be like, ‘oh, I don’t know,’…We always try to keep things to ourselves.’(Roshan*, Male, 50–59 years)
G. Research staff	‘This sort of study, it may be more inclined to ask quite personal questions about health, our diet, what we eat, and in our culture…there may be a sort of stigma behind it and judgment [from research staff]. So I think that might stop people from participating in it if they don’t know what sort of questions are asked, whether they’ll be judged for what they do, eat and drink’ (Heneesha*, Female, 18–29 years). *‘It depends on the confidentiality that you build, because sometimes we may feel that you will misuse our data, and also sometimes we may feel that we will be blamed if, having some bad kind of behaviour. So, providing more confidentiality, building their confidence regarding the privacy of the data will improve providing you with all personal details and lifestyle factors.’* (Nilushi*, Female, 30–39 years)
H. Australian society	*‘I just hope that if this research is published, employees may not want to employ South Asian people because they’ve got a higher degree of health problems, which means there are more absent days, employment risk, etc…Even though you could say, most South Asians…they’ve contributed a lot. But that will go by the wayside because we are still- like I’ve always seen this sort of ceiling…But I think in Australia there’s a great bias towards people of colour, too.’* (Doreen*, Female, 50–59 years). *‘I remember I went to a party of my kids… and the person sitting next to me, Australian, and I said to him that I am working on some immigrant health specialty, and he said, ‘Who cares? They came here on their own choice,’ and I was surprised, [laughs] … South Asians are basically, if you see the literature, they are identified as, you know, they either have good—they especially emphasise on education, achieving a good career, so you are not taking someone who is useless for your community. Everyone is useful.’* (Nadia*, Female, 30–39 years).
I. Insurance companies	*‘The Facebook groups people are sending me, you are on a hidden agenda, you want to increase the insurance fees of South Asian immigrants.’* (Nadia*, Females 30–39 years) *‘One of the things that came up in my mind was, what if insurance companies used it? And we didn’t get health insurance?’* (Neela*, Female, 40–49 years)

^*^pseudonyms provided in quotes

### Negotiating cultural identity, diversity, and belonging within the South Asian diaspora

The theme ‘cultural connection’ encompasses the cultural factors and perspectives of South Asian communities that can influence genomic research participation (Table 3). Views towards involvement in health and genomic research were impacted by one’s cultural heritage and identity, and community perceptions.

For most participants, cultural connection was a main driver of research participation, with many individuals noting they would be less willing to participate in genomic research that was not geared toward South Asian communities (Table 3A). However, several participants emphasised the importance of researchers understanding the unique and shared perspectives of the South Asian diaspora, which can vary in cultural practices, religion, spoken language, and environment (Table 3B). Such differences were noted to impact community engagement and the strategies needed to support research participation. Communication and building rapport through language was frequently expressed, including the importance of conversing in the community’s native language and translating documents into multiple languages (Table 3C). Some participants noted generational differences in language preferences, explaining that their parents would be interested in participating if information had been made accessible in their language (Table 3C). However, it was acknowledged that it would not be possible to translate documents into all South Asian languages and having varied strategies, including translated documents into some languages (e.g. Punjabi, Urdu, Bengali, and Hindi), coupled with access to interpreters, would improve research participation.

Early discussions with community leaders and CAG members, pre-focus groups, suggested understanding if South Asian participants had issues with blood collections due to cultural or religious reasons. In our participant cohort, providing a biological sample, such as a blood test, was not seen as a concern or barrier to research participation (Table 3D).

The complexities of migration, including the associated loss of identity and connection to family history, were noted by most participants. Occasionally, the complexities of identity and identity-loss were connected to participants’ engagement with this research, where some had sought to connect with culturally similar groups (Table 3E). These issues were particularly pronounced among individuals of Indo-Fijian heritage who often faced challenges of misclassification in Australian society. However, these challenges further motivated individuals to engage in genomic research as an opportunity to gain knowledge, enhance cultural connections and help future generations.

Fear and the potential for stigma and discrimination were identified as significant determinants of research participation, which was prevalent across all focus groups. Such issues were multifaceted, stemming from historical mistreatment, challenges from migration, and cultural norms. Community discrimination was noted, with many individuals commenting on the negative views towards health conditions from within South Asian communities that could hinder marriage and social standings of affected or at-risk individuals and/or their relatives (Table 3F). Similarly, while many participants felt positive towards attending community-facing events, others preferred more discrete methods of participation due to the need for privacy and fear of judgment (Table 3F). Potential stigma from the research staff was also raised, arising from a lack of cultural awareness (Table 3G). On a societal level, fear of personal and group harm was noted in all focus groups, which were commonly driven by personal experiences of racism or prejudice (Table 3H). At times, participants reflected on feelings of worth, justifying their role and belonging in society (Table 3H). Some participants also questioned the impact that genetic data could have on insurance, with fears noted about how this information could be used to raise premiums for South Asian immigrants (Table 3I). However, misunderstandings regarding the use of genetic risk information in health insurance in Australia were common, which is community-rated, and therefore premiums are not impacted by health risk (Table 3I).

### Trust and relationship-building

The third theme of ‘trust and relationship-building’ encompasses the significance of trust, establishing trustworthiness, and relationship-building with communities to support genomic research engagement. Here we define trust as the reliability, belief and truth from an individual, while trustworthiness is the ability to be relied on and believed to be truthful [27].

Trust was a key factor in research-related decisions among all participants, which mitigated fears and concerns described. Participants frequently recommended approaching community leaders, registered community organisations who are trusted sources of information (Table 4A). In fact, when reflecting on their decision to attend the focus group, most individuals described being told of the study by trusted connections, such as friends, colleagues or community members, which helped them feel reassured about participating (Table 4B). Such feelings were enhanced by perceptions of The University of Queensland as a trustworthy and credible institution (Table 4C). Many participants voluntarily shared details about trusted groups and organisations to assist in disseminating research information, along with events and places where a high volume of South Asians access and attend, such as religious and spiritual gatherings (Table 4D). These suggestions were community-specific and differed across the various ethnolinguistic groups present in our cohort.

**Table 4:** Representative quotes for theme Trust and relationship-building*.

Sub-theme	Quote
**Trust and trustworthiness**	
A. Trusted community leaders and champions	‘…About engaging community leaders, I think that would really, really help to get the message out in a way that the local community understands with someone that they trust as well, because sometimes establishments and different things, it’s hard….’ (Heneesha*, Female, 18–29 years). ‘*Being a community leader, it really helps because a lot of people do follow us, and they do listen to us, and they do respect us… the best bet is to get hold of the community leaders because they are the ones who are leading their community, and then that will pass that information down*.’ (Roshan*, Male, 50–59 years). *‘A lot of the South Asian group go to South Asian doctors, if they tell them ‘Hey there’s this research going on, you can participate’ and it’s benefit for both of them*…’ (Irfan*, Male, 30–39 years).
B. Trusted connections	*‘I was invited by [friend]. [He] actually briefed me about the purpose of this study. And so far, I have understood that there is a lack of representation from South Asian samples, our population, in the genomic studies conducted in the Western world. So I thought, why not? We should contribute to this space. That’s why I’m here*.’ (Hamza*, Male, 30–39 years) ‘By word of mouth, I think, we still are like believing more when my friend would tell me something than see it on a billboard or something’ (Irfan*, Male, 30–39 years).
C. Institutional trustworthiness	‘Because you remove the UQ sign from there, I’m not participating’ (Sakshi*, Female, 25–29 years) *‘The fact this was again linked to UQ and from someone from UQ saying that it’s legit’* (Saleem*, Male, 30–39 years).
D. Engagement via community events	*‘Religious places like gurdwaras, because every Sunday there are a lot of people in this particular* gurdwara [Sikh place of worship]*…I visit often in gurdwaras. There is nothing like that, ‘this is the only place for one community’. That* [the gurdwara] *is for all the people.’* (Ajeet*, Male, 30–39 years). *I know people in the South Asian communities are very tight, and I think reaching out to specific events that are for them is really a very good opportunity to talk about these things*. (Heneesha*, Female, 18-29 years). ‘*Religious communities obviously, because of the very nature of how community exists, might be a good way to get outliers in communities, so local mosques*, mandhir [Hindu place of worship].’ (Saleem*, Male, 30–39 years).
E. Data privacy	*‘…when someone goes for research, one of the protective mechanisms is…the trustworthiness. Will it* [data access] *be open to all?I understand that many of the genetic research data are open on many platforms… [do they] have a proper policy for maintaining the privacy and confidentiality?*’ (Zaheer*, Male, 30–39 years)
**Relationship building and representation in the research team**	
F. Researcher representation	‘*It’s good to see more female South Asian researchers, so yeah, I was like, okay, I’ll support them*.’ (Yamuna*, Female, 30-39, Indian) ‘*I felt very at ease in being able to discuss my thoughts and experiences without concern. It was also great to have people from a similar background who understood the nuances of the topic.’* (Trisha*, Female, 30–39 years).
G. Feeling heard	‘*It is important that we are heard, even in health-related matters.Therefore, even if some of us, only a few of us, are coming forward, it is a signal that this is an important matter, and we need to support each other*.’ (Karthik*, Male, 70+ years).
H. Ongoing relationships	‘I feel like having the trust is built by people that you’ve been communicating with… And so, to have a person that you can keep that sort of rapport going throughout this stage that would definitely be helpful… it would just be good to establish a continuing relationship with the people that are involved in this study, because it gives you a little bit of an understanding of why we're all here. What are we putting our efforts towards? What is the legacy that we’re going to be leaving? It just gives a little bit more gravitas to this study.’ (Dharsha*, Female, 30–39 years).
I. Acknowledgment and empowerment	‘*I just want to say, thanks for this. It’s a really useful project, and I’m really happy that you guys are doing it*.’ (Selvi*, Female, 50-59 years). ‘The focus group was empowering and enlightening. Right from the start, the conversations were engaging, and the researchers provided us with lots of support and respect. They also acknowledged the apprehensions that are usually associated with providing sensitive health information. Overall, I felt proud to have played a small part in this very important study, and I hope it continues to gain traction as it progresses.’ (Written feedback, deidentified)

^*^pseudonyms provided in quotes

Participants frequently emphasised the importance of establishing ongoing relationships and having researchers who understood community perspectives. For many, representation of South Asian individuals in the research team enhanced trustworthiness of the study, which helped them feel more at ease in sharing their perspectives without fear of judgment (Table 4E). Authentic relationship-building involved feeling seen and transparent communication, which further encouraged discussions, increased awareness, and active community involvement (Table 4F). This was particularly important around discussions that involved stigma and trust. Similarly, while concerns about data privacy were frequent, these were mitigated by researcher trustworthiness (Table 4E).

A trusting relationship was seen as essential in developing genuine partnerships with communities to support genomic research participation. This relationship-building was reflected by many study participants who expressed appreciation for the ongoing communication and engagement with the SAGHA research team and stated the importance of continuing that relationship even after the research was completed. For many, this was an important aspect of relationship-building that included ongoing researcher engagement, having a single point of contact and two-way communication (Table 4H). Several participants commended the research team and recognised the efforts in their active work with the community as partners. Feedback received from participants during and following focus groups indicated that they felt empowered by being involved, appreciated being able to contribute to the study, and felt understood by the researchers who were familiar with community values, views, and context (Table 4I).

## Discussion

This study aimed to identify the attitudes and perceptions of Australian South Asians towards genomic research, with the goal of informing the development of a culturally tailored recruitment protocol. In line with CBPR and frameworks in genomic research [2, 6, 8], defining and understanding the context of the research is an essential first step to enhancing engagement and establishing sustainable long-term relationships. Overall, our participants reported positive views about genomic research, with all individuals recognising benefits for themselves, their families, and/or their community. However, most participants had limited knowledge of genomic research pre-study enrolment. The education provided as part of the study, along with the majority of South Asian-led researchers and representation from community leaders, was a key driver in increasing support for research participation. The strong lived experience of CVD, motivation to improve representation for the community, migration, and prior experience with health inequities further increased interest.

In Australia, most of the South Asian population has migrated under a student or Skilled Migration Program, with Indians making up the largest source of migrants under these visa schemes [23, 28]. This migration context is important for understanding our participant cohort, which was predominantly comprised of first-generation migrants. Although health literacy was not formally assessed, the high interest in research and the ease with which participants understood the concepts discussed were likely reflective of their generally high levels of education [29, 30]. However, our participants also acknowledged the diversity within the South Asian diaspora and noted that interest in research and subsequent approach to recruitment would need to vary between subgroups, including across generations and migration status (e.g. parents of skilled migrants and Australian-born South Asians). Future research should explore diverse views across broader sociodemographic perspectives to capture additional factors that may impact research participation.

Trust, relationship-building, and perceived trustworthiness were central to research engagement within our cohort. These were largely driven by concerns around stigma, discrimination and lived experiences of racism or prejudice in Australia. Such experiences are not unique to our cohort [31], with a survey of Indian Australian migrants identifying that approximately 50% of participants had experienced frequent discrimination [32]. Such findings have implications for future genomic research with this population, including a need to prioritise long-term and sustained relationship-building with each community. As shown with this project, trust and trustworthiness are active processes and can be achieved through inclusion of community champions in the research, racial concordance within the research team, involvement of community leaders, transparency and open communication [27, 33, 34]. Furthermore, providing tangible community benefits, such as health education and improved health outcomes, together with transparency of research processes, are essential to driving community partnerships, fostering trust, and establishing trustworthiness [27]. While such efforts have contributed to meaningful engagement, it is important to acknowledge that current funding cycles are not conducive to sustaining long-term community relationships [10]. Thus, novel ways to support long-term community engagement are needed, which will require a systemic approach across government, university, researchers, and individual levels. Collectively, our findings underscore the importance of community partnership and honouring community knowledge to establish meaningful relationships and subsequently improve diversity of research.

Our findings should be interpreted in line with the study limitations. Firstly, focus groups were advertised and conducted in English, and could not accommodate unanticipated translation assistance. While the participant sample was broadly representative of the Australian South Asian population [35], limited differences were observed in perspectives across ancestry and ethnic groups. This finding may reflect the relatively homogenous nature of the cohort, particularly in relation to migration background and education level. Nevertheless, participants identified how views may differ across generations, education and other sociodemographic factors. The study also focused on the context of CVD, and therefore, attitudes towards research participation for other health conditions may vary. The strengths of this study included the relationship building and community engagement via a research team comprised of individuals of South Asian ancestry, and the establishment of a CAG. These strategies facilitated recruitment and supported a safe, respectful environment conducive to sharing cultural perspectives, views, and experiences. Overall, findings will have implications for community engagement in genomic research, and these community perceptions will inform the next phase of the SAGHA study, including the development of an inclusive framework for conducting genomic research within Australian South Asian communities.

## Supplementary information

Focus group presentation

Consortia membership

## Data Availability

The datasets generated during and/or analysed during the current study are not publicly available to maintain participant privacy and confidentiality, but are available from the corresponding author on reasonable request.
