# Correction: *“There’s no representation”*: a qualitative study of attitudes and motivations towards genomic research participation among Australian South Asians

**DOI:** 10.1038/s41431-026-02157-z

**Published:** 2026-06-23

**Authors:** Vaishnavi Nathan, Heena Akbar, Aideen McInerney-Leo, Deborah Gilroy, Anjali Henders, Reema Naresh, Nahid Choudhury, Maleeha Waqar, Sonia Shah, Tatiane Yanes

**Affiliations:** 1 Frazer Institute, The University of Queensland, Dermatology Research Centre, Brisbane, QLD Australia; 2 School of Public Health, Faculty of Health, Medicine and Behavioural Science, The University of Queensland, Brisbane, QLD Australia; 3 Institute for Molecular Bioscience, The University of Queensland, Brisbane, QLD Australia; 4 Brisbane Northside Fiji Senior Citizens, Brisbane, QLD Australia; 5 Metro North Mental Health, School of Health Sciences, Department of Nursing and Allied Health, Swinburne University of Technology, Melbourne, VIC Australia; 6 School of Nursing, Queensland University of Technology, Kelvin Grove, QLD Australia; 7 School of Biomedical Sciences, The University of Queensland, Brisbane, QLD Australia; 8 The University of Queensland Faculty of Health, Medicine, and Behavioural Sciences, Cardiology Department, Royal Brisbane and Women’s Hospital, Brisbane, QLD Australia; 9 Department of Cardiology, Royal Brisbane and Women’s Hospital, Brisbane, QLD Australia; 10 Sunshine Coast University Hospital, Birtinya, QLD Australia; 11 Genetic Health Queensland, Royal Brisbane and Women’s Hospital, Herston, QLD Australia; 12 School of Biomedical Sciences, Faculty of Health, Queensland University of Technology, Brisbane, QLD Australia; 13 QIMR Berghofer, Brisbane, QLD Australia; 14 General Practice Clinical Unit, Medical School, The University of Queensland, Brisbane, QLD Australia

**Keywords:** Genetics research, Psychology

Correction to: *European Journal of Human Genetics* *“There’s no representation”*: a qualitative study of attitudes and motivations towards genomic research participation among Australian South Asians

10.1038/s41431-026-02129-3; Article published online 21 May 2026

In the original article, Sonia Shah should have been denoted as a co-corresponding author.

The original article has been corrected.

