# Supplementary material for: “There’s no representation”: a qualitative study of attitudes and motivations towards genomic research participation among Australian South Asians
Source: Eur J Hum Genet. 2026 May 21;34(8):1087–96. doi: 10.1038/s41431-026-02129-3 (PMC13424584; doi:10.1038/s41431-026-02129-3)

The University of Queensland
Australia
Create Change

Focus Group:
The South Asian Genes and Health in Australia (SAGHA) Study

Acknowledgement of Country

The University of Queensland (UQ) acknowledges the Traditional Owners and their custodianship of the lands on which we meet.

We pay our respects to their Ancestors and their descendants, who continue cultural and spiritual connections to Country.

We recognise their valuable contributions to Australian and global society.

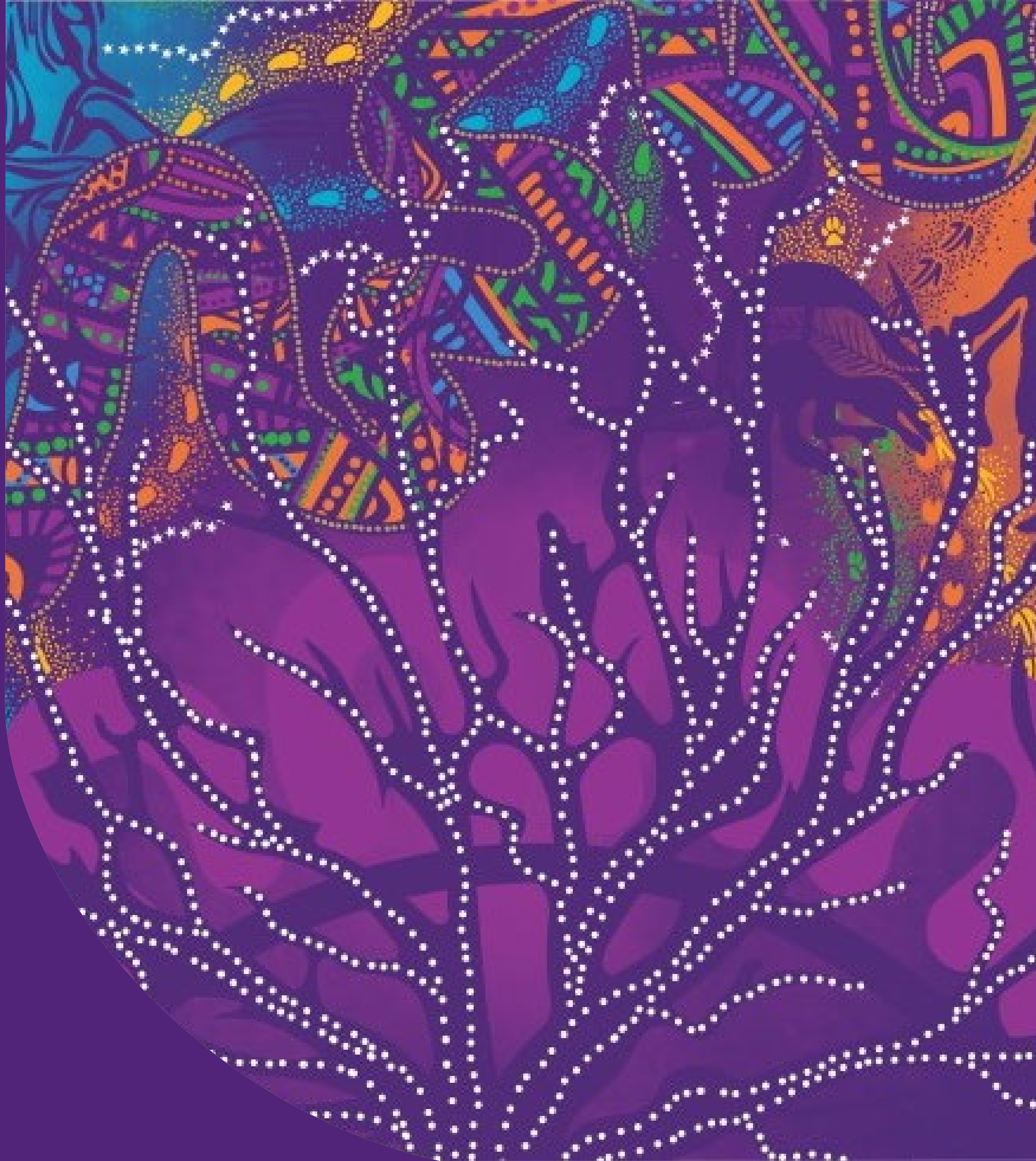

OUTLINE

01

Introductions

02

Genetics presentation

03

Genetics and heart disease

04

Conclusion

Introduction: Who are we?

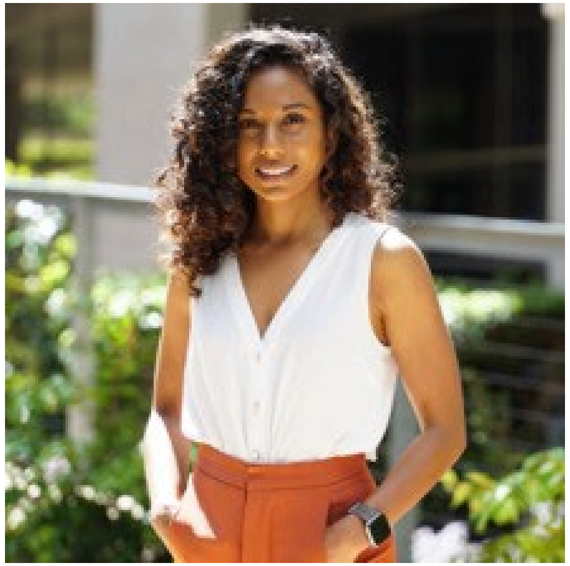

Sonia Shah
Project Lead
Researcher, UQ

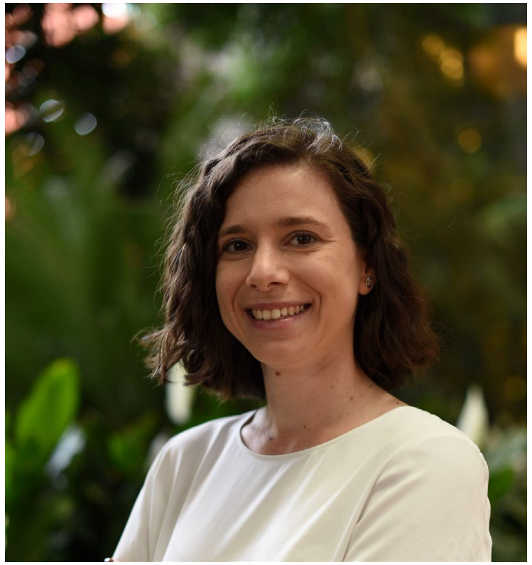

Tatiane Yanes
Genetic Counsellor
Researcher, UQ

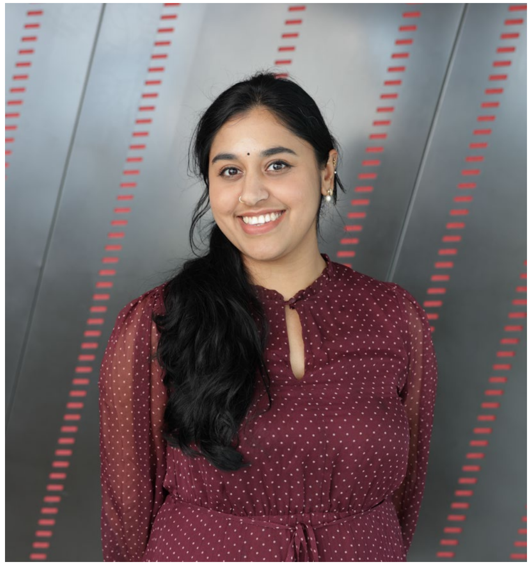

Vaishnavi Nathan
Genetic Counsellor
Researcher, UQ

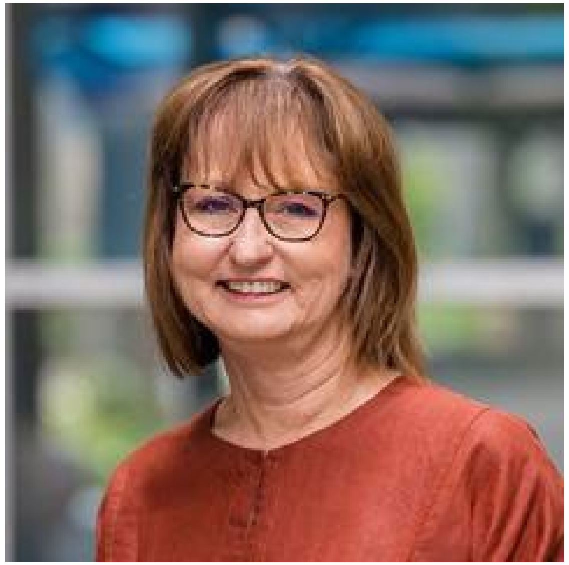

Deb Gilroy
Senior Project Officer
UQ

Why is health-related research important?

- Understand cause and risk factors for disease
- Test new treatments and vaccines
- Determine which medications work best
- Identify any side-effects of medication
- Identify groups of individuals who are more vulnerable to disease
- What criteria should be used to start medication

Improve healthcare and public health

Heart disease

- Leading cause of death in Australia
- 8.7% of healthcare expenditure ($11.8 billion) attributed to CVD
- Both our genetics and lifestyle play a role
- The right intervention at the right time can prevent disease

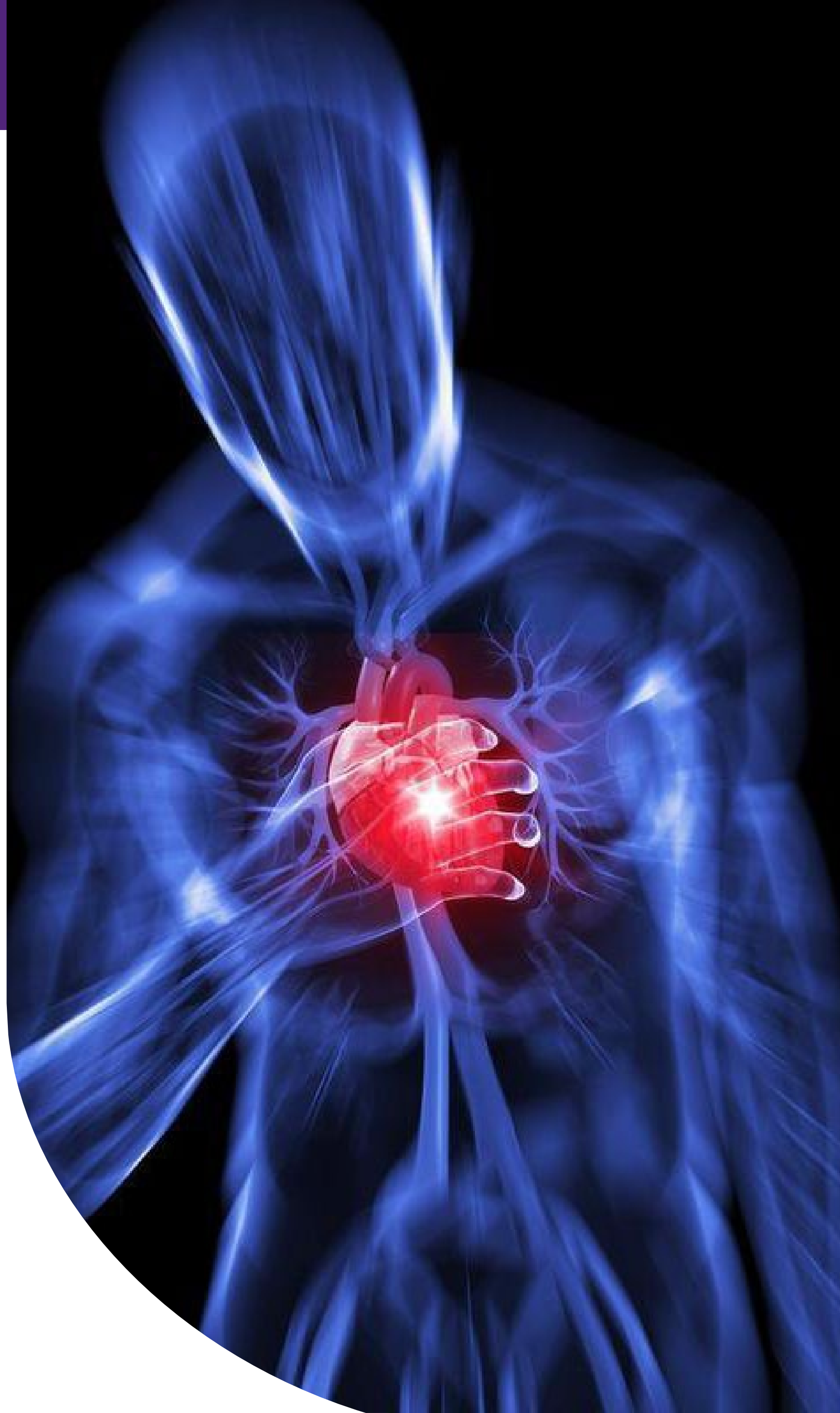

South Asian are more likely to get heart disease

- South Asians are **2-times** more likely to get heart disease
- 25% of heart attacks occur in under 40 yrs, and 50% occur in under 50 yrs
- Develop disease up to 10 years earlier than the general population, on average (statistics from https://stanfordhealthcare.org/)

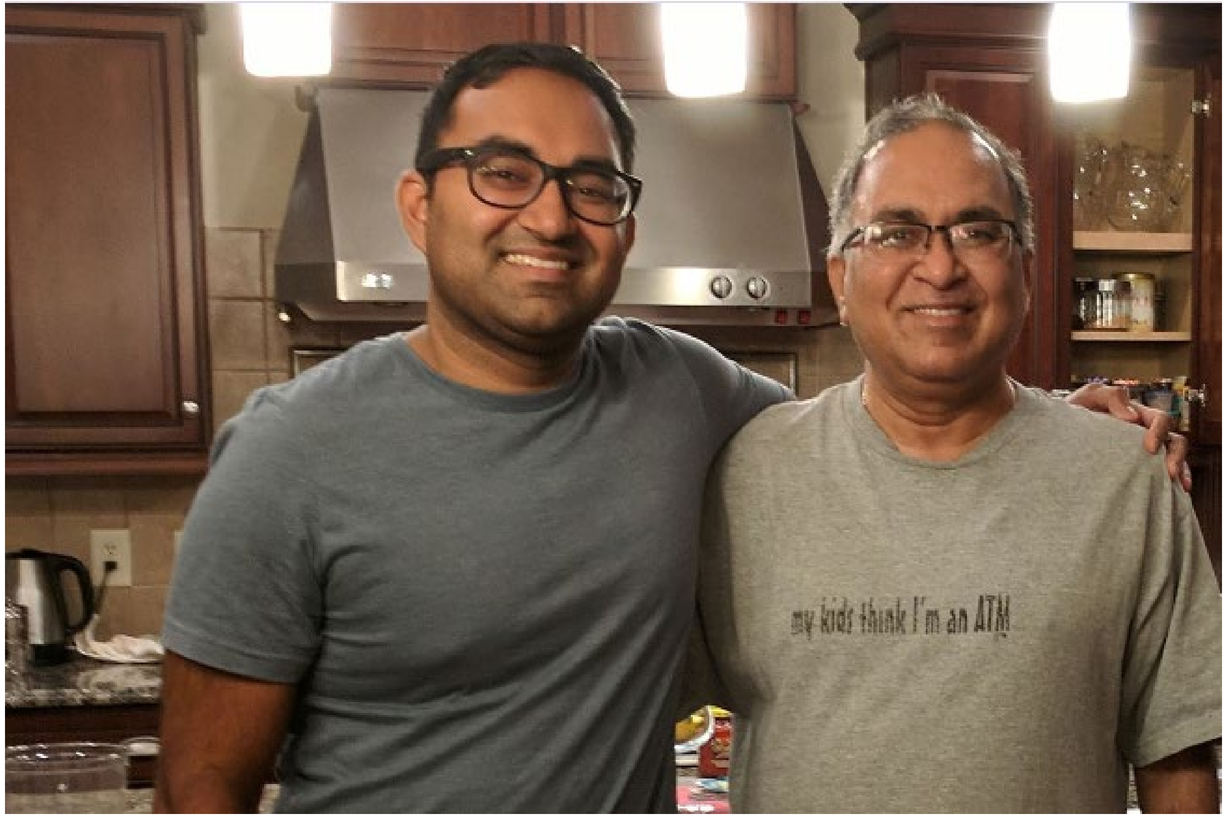

Adi's Story:

- Father diagnosed with Type 2 diabetes

- Changed his diet, was more active (walking everyday for 1 hour) and took medication

- 2 years after the diagnosis he died of heart disease (Adi was in his mid-20s at the time)

https://www.acc.org/Latest-in-Cardiology/Articles/2019/05/07/12/42/Cover-Story-South-Asians-and-Cardiovascular-Disease-The-Hidden-Threat

Preventing heart disease - Australia's Heart Health Check

Heart Health Check: **risk** of developing heart disease within 5 years

Traditional risk factors for heart disease

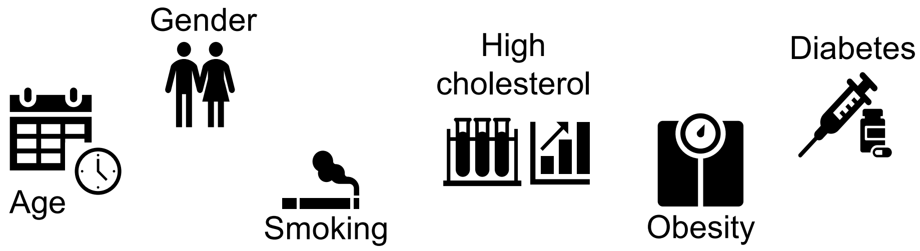

Preventing heart disease - Australia's Heart Health Check

BUT…

- Does not consider ethnic differences in heart disease risk

- The heart health check used in Australia **under-estimates** risk in South Asians

Framingham Heart Study

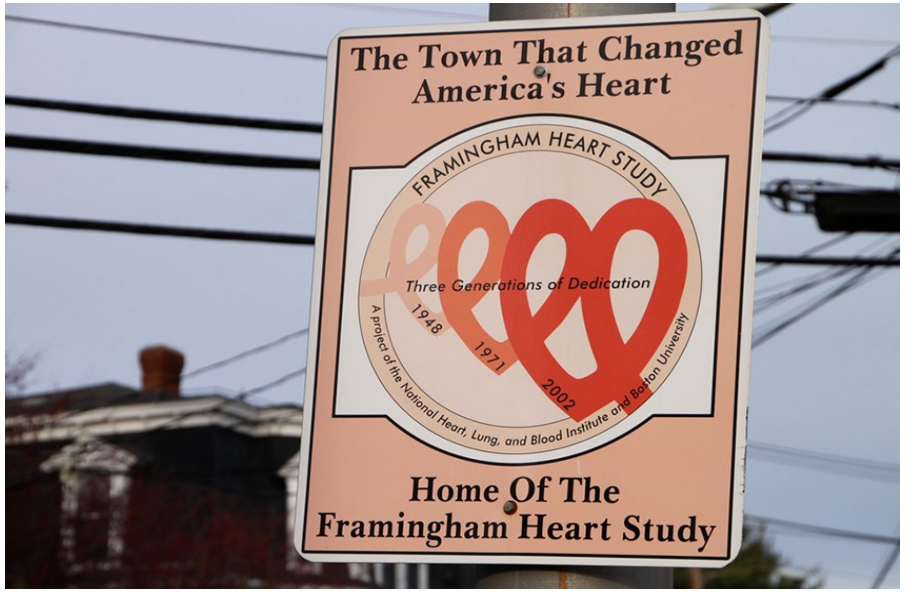

Image Credit: https://www.bostonmagazine.com/

Preventing heart disease - Australia's Heart Health Check

BUT…

- Less accurate in younger adults and those without traditional risk factors

- 20% of first-time heart attack patients have no traditional risk factors

Heart attacks more likely to be fatal among people with no risk factors, new research finds

By national medical reporter Sophie Scott and the Specialist Reporting Team's Mary Lloyd

Posted Wed 10 Mar 2021 at 9:32am, updated Wed 10 Mar 2021 at 2:33pm

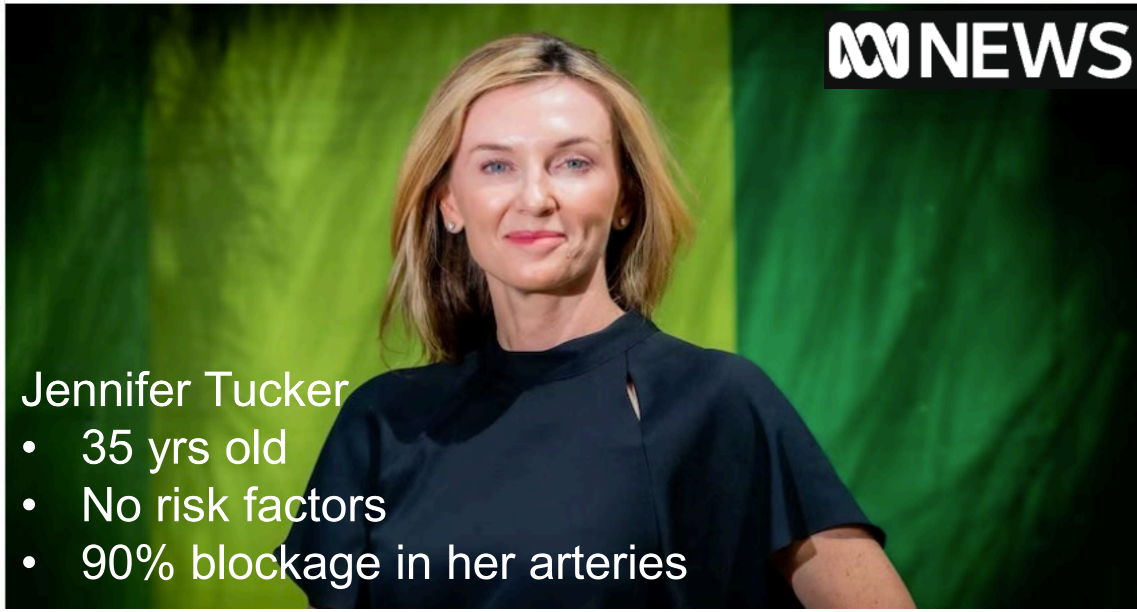

Genomic information can improve risk calculation

- **Genomics is the study of our DNA** e.g. How differences in our DNA affect our health

- Our genetic risk is there from birth - if known, we can make the right lifestyle choices early on in life to prevent disease.

Government investment of $500 million over 10 years in genomic research (2018 - 2028)

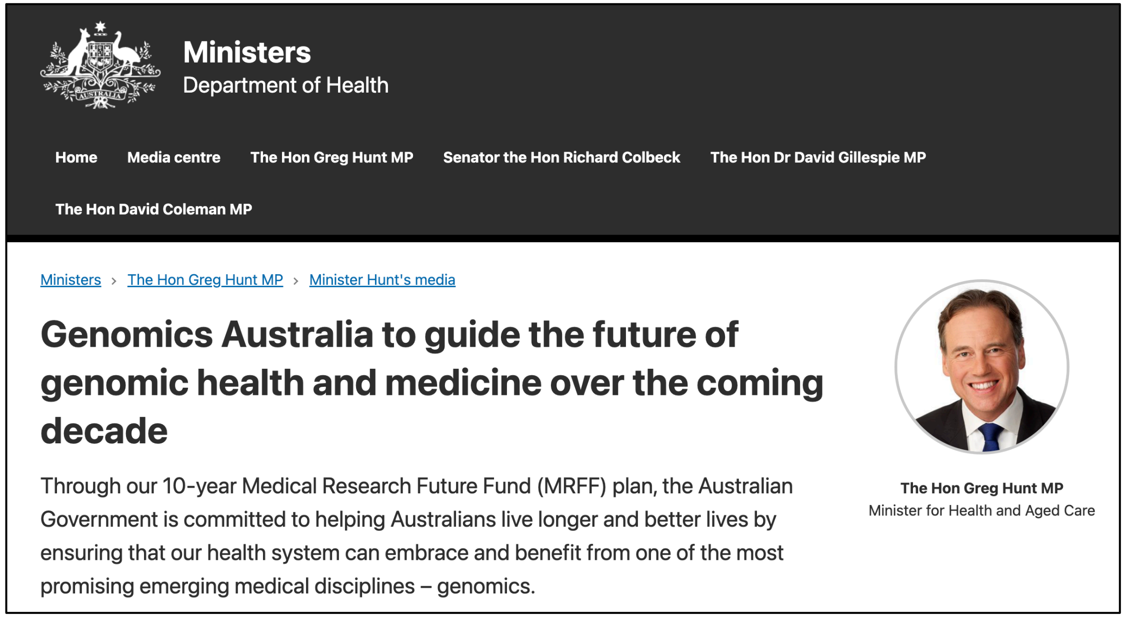

Ministers
Department of Health

Home · Media centre · The Hon Greg Hunt MP · Senator the Hon Richard Colbeck · The Hon Dr David Gillespie MP · The Hon David Coleman MP

Ministers › The Hon Greg Hunt MP › Minister Hunt's media

Genomics Australia to guide the future of genomic health and medicine over the coming decade

Through our 10-year Medical Research Future Fund (MRFF) plan, the Australian Government is committed to helping Australians live longer and better lives by ensuring that our health system can embrace and benefit from one of the most promising emerging medical disciplines – genomics.

The Hon Greg Hunt MP
Minister for Health and Aged Care

Genomics in health care

Information about a person's genome is already being used to:

- Identify and diagnose rare genetic disorders (e.g. in newborn screening)
- Guide treatment choices in cancer
- Predict a person's response to treatments, so doctors can provide the best treatment option for a person

Current research: Using genetic information in heart health checks

Genomic research today will inform healthcare tomorrow…BUT…

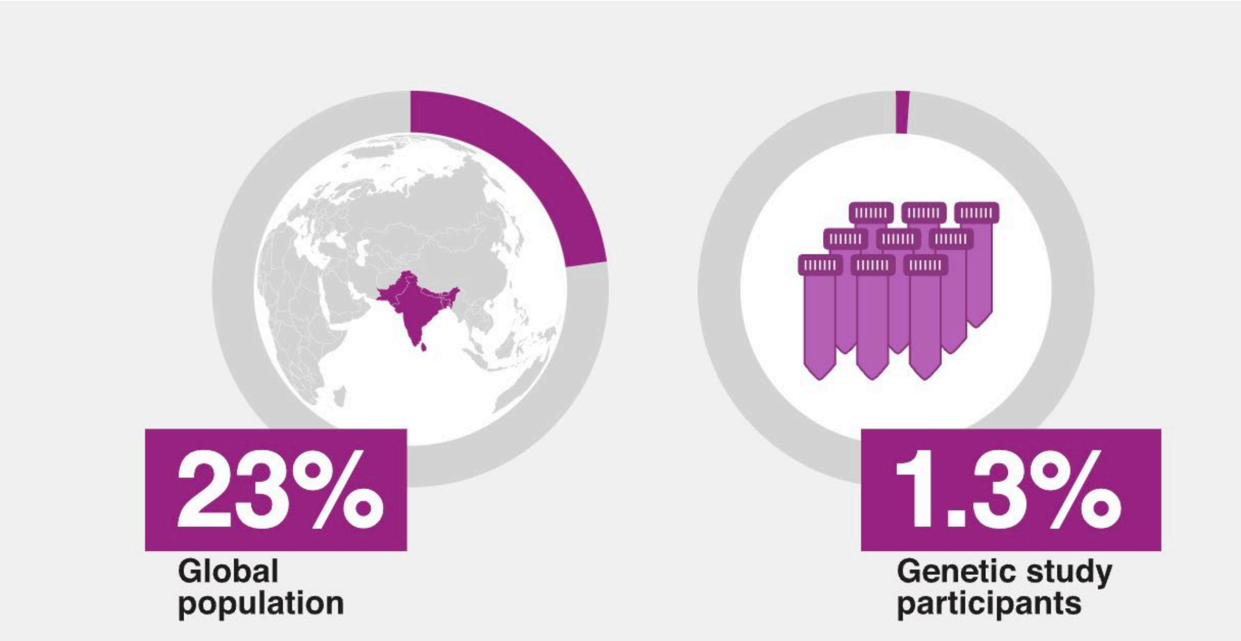

Image from ourhealthstudy.org

Australia's medical guidelines are based on research done in mostly European ancestry individuals

Data from genetic studies:

- 86% participants are of European descent
- 1.3% participants are of South Asian descent

Why is it important that the South Asian community is represented in genomics research?

Improve healthcare for future generations of South Asians

- Reduce heart disease burden in the South Asian community
- Ensure health research considers **all** Australians

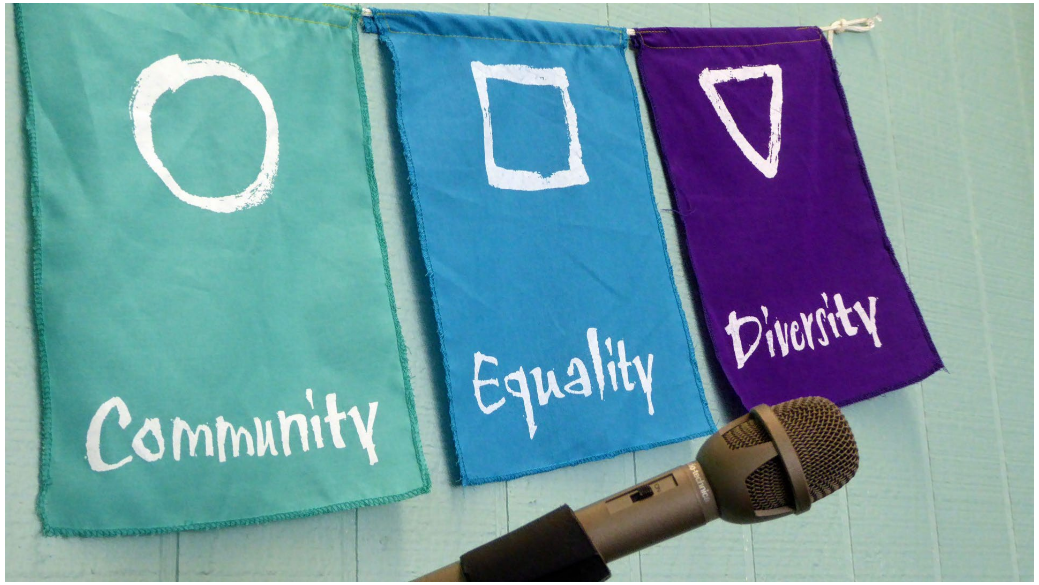

International efforts

East London Genes and Health Study (Launched 2015)

50,000 British Bangladeshi and British Pakistani participants

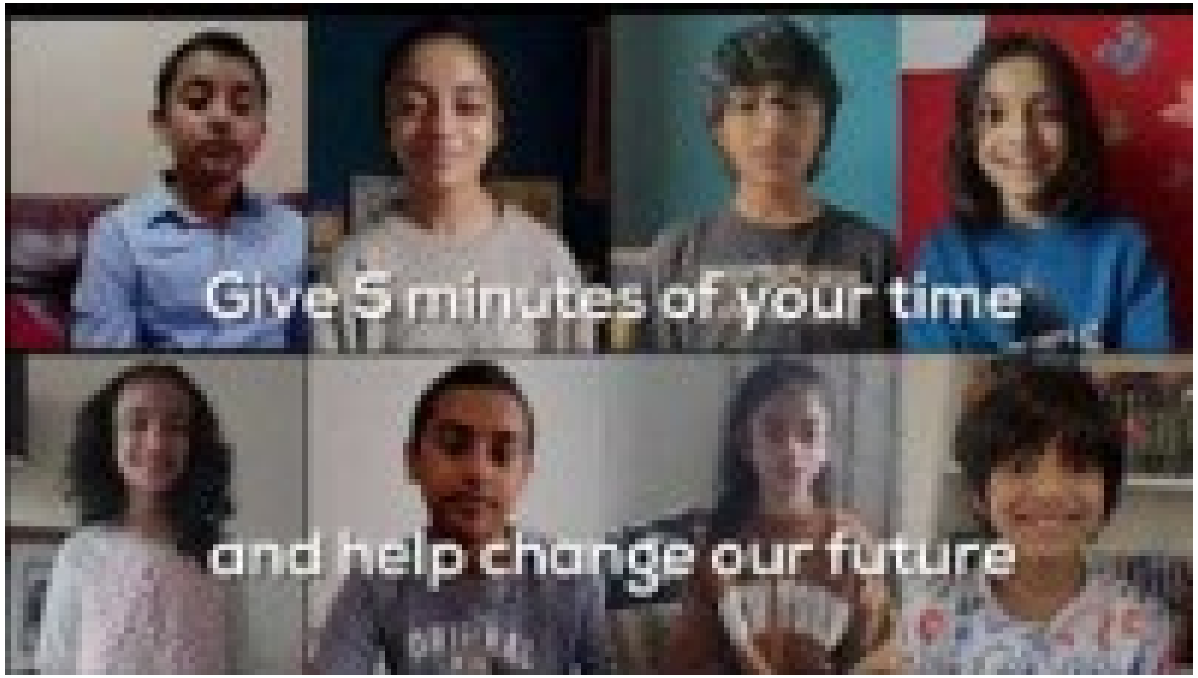

Our Health Study (Launched 2022)

- US population
- Aiming to recruit 10,000 South Asians

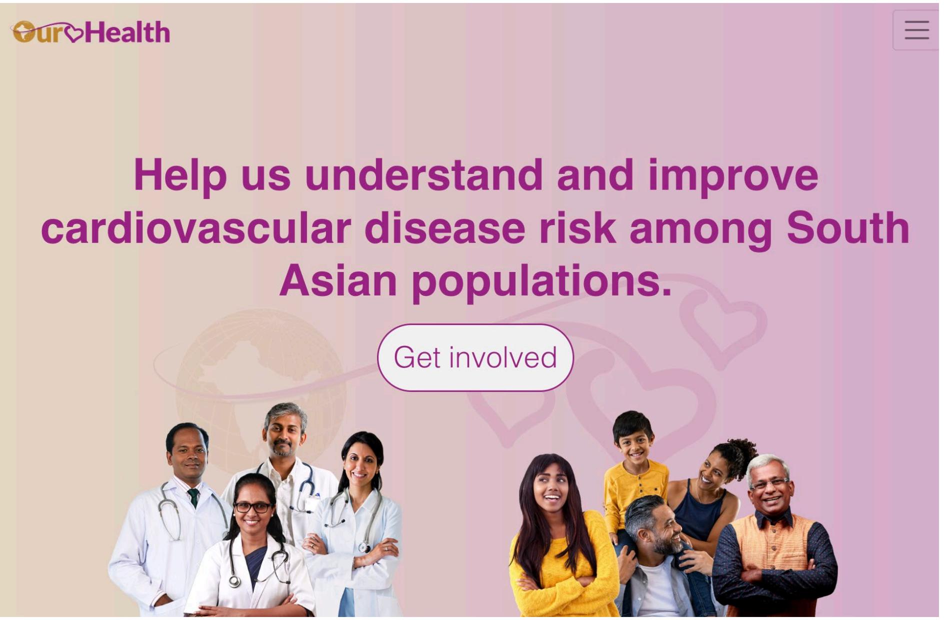

Australia's first ever genomic study focused on South Asians

STAGE 1 (2023) → STAGE 2 (2024)

Aim 1: Focus Group Discussions

What prevents South Asians from participating in health and genomic research and how to increase participation

→

Aim 2: Pilot Study Recruitment

Recruit 200 individuals from the Queensland South Asian community

- Health questionnaire
- Blood sample for DNA,
- Blood pressure, glucose and cholesterol measures

→

Aim 3: Heart disease risk assessment of pilot study participants

Heart Health Check risk
Genetic risk

Long-term goal: improve medical guidelines around heart disease for South Asian Australians

Introduction: Purpose of these focus groups

- High impact for South Asian population
- Voices from the South Asian community
- How can we best work with the South Asian community to **improve participation** in genetics research?
- Your discussions go forward to creating a framework

Housekeeping

Respect opinions

Private and confidential

Recording

Open Discussion

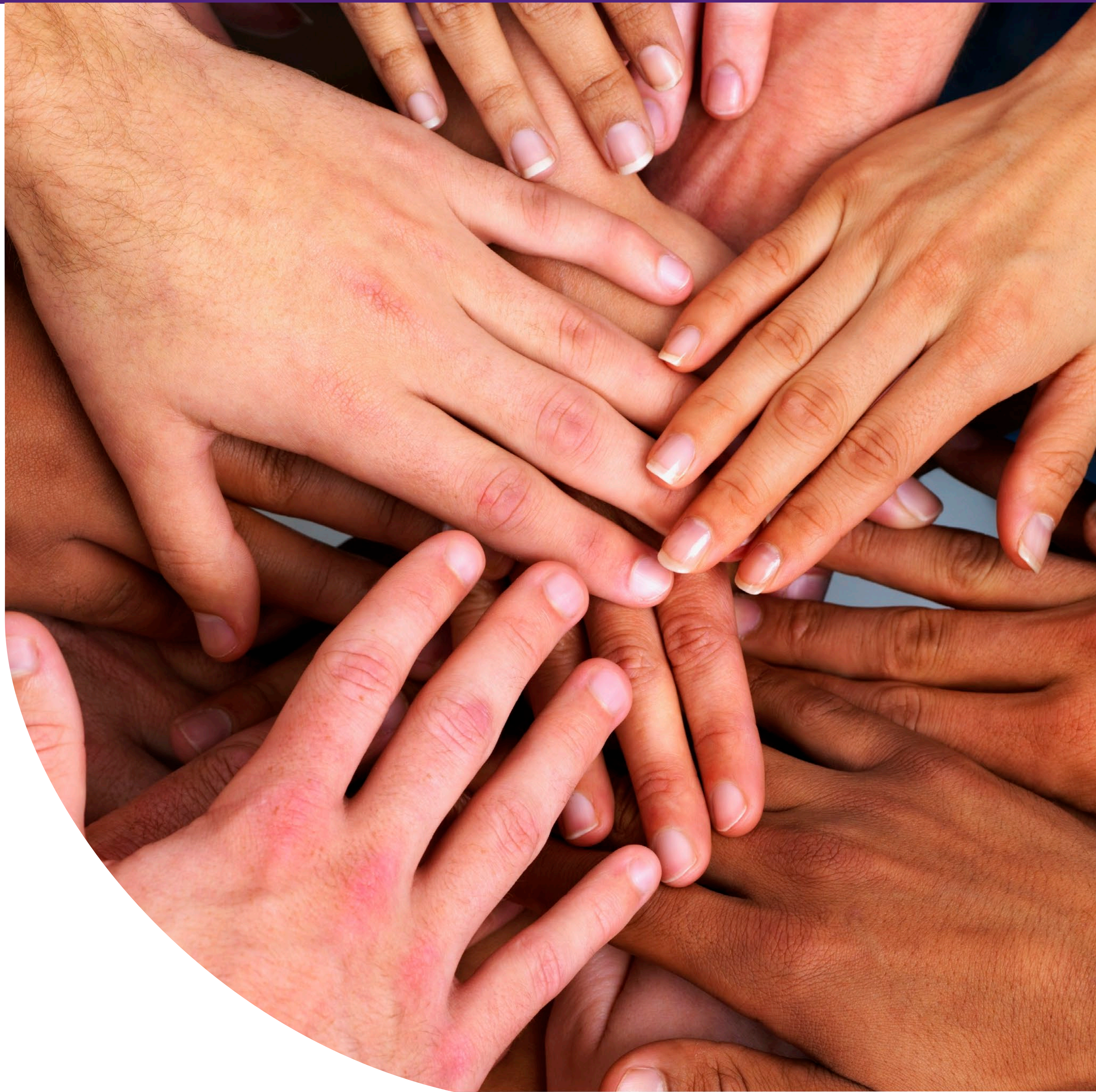

Introduction: Participants

1. **Can you please tell me about yourself?**
    - What sort of work do you do?
    - Tell me about your family?
    - What is your family ancestry?

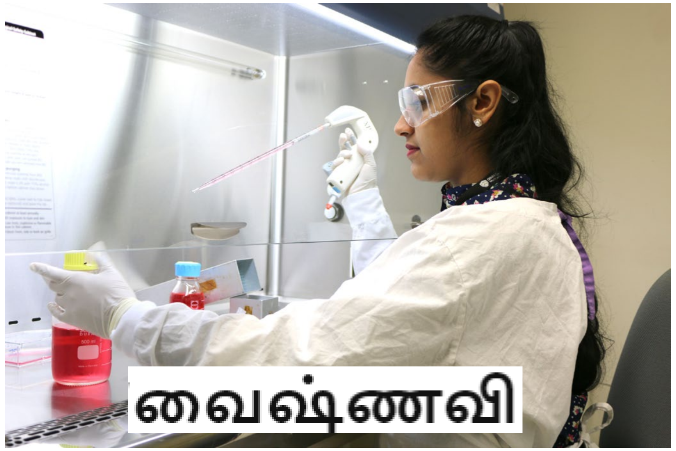

Introduction: Participants

2. Have you, or anyone you know has ever been part of any health research?

OUTLINE

01

Introductions

02

Genetics presentation

03

Genetics and heart disease

04

Conclusion

Question

3. What do you know about genetics?

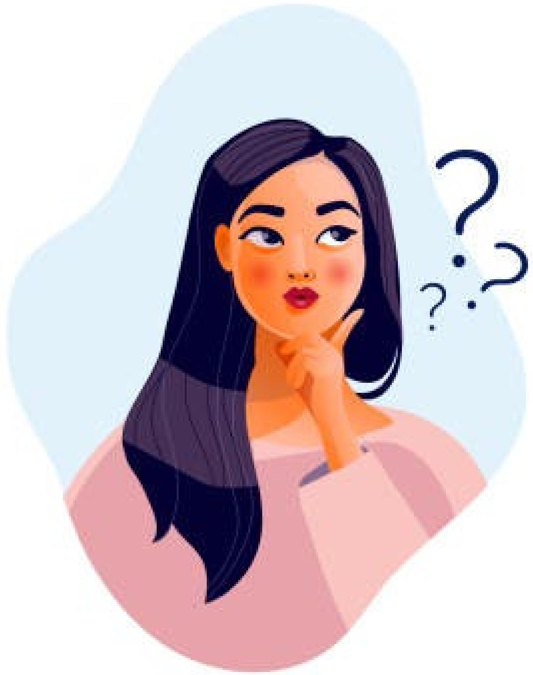

Genetics

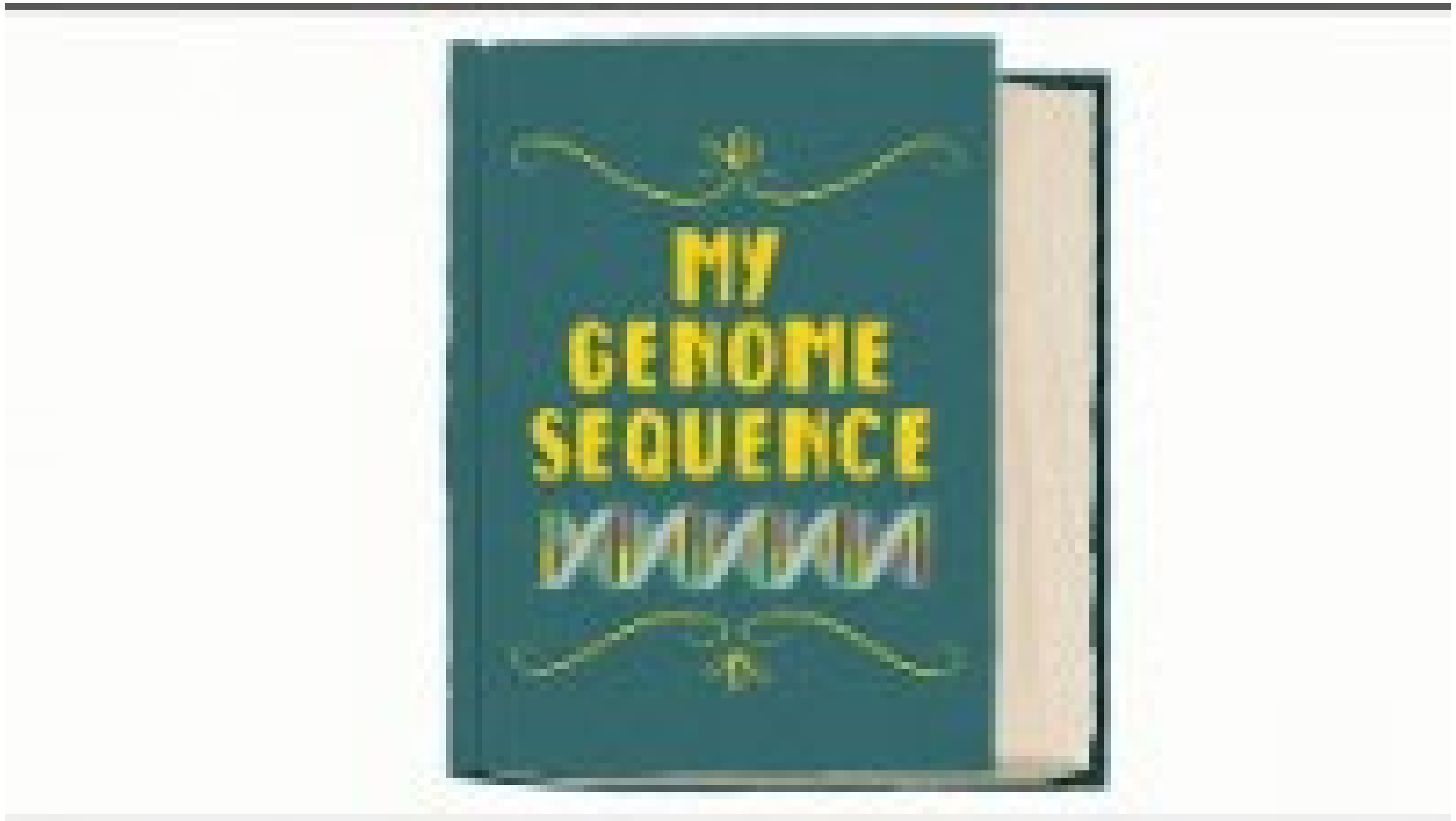

Question

4. What are your thoughts about participating in genetic research?

Question

5. **What might make you more or less inclined to participate in genetic research?**

OUTLINE

01
Introductions

02
Genetics presentation

03
Genetics and heart disease

04
Conclusion

Cardiovascular disease (CVD)

- Leading cause of death globally and in Australia
- CVD umbrella term including heart, stroke and blood vessel diseases
- Common condition, affecting more than 4 million Australians currently
- South Asians have higher prevalence and earlier age of onset
- Genetics AND environment

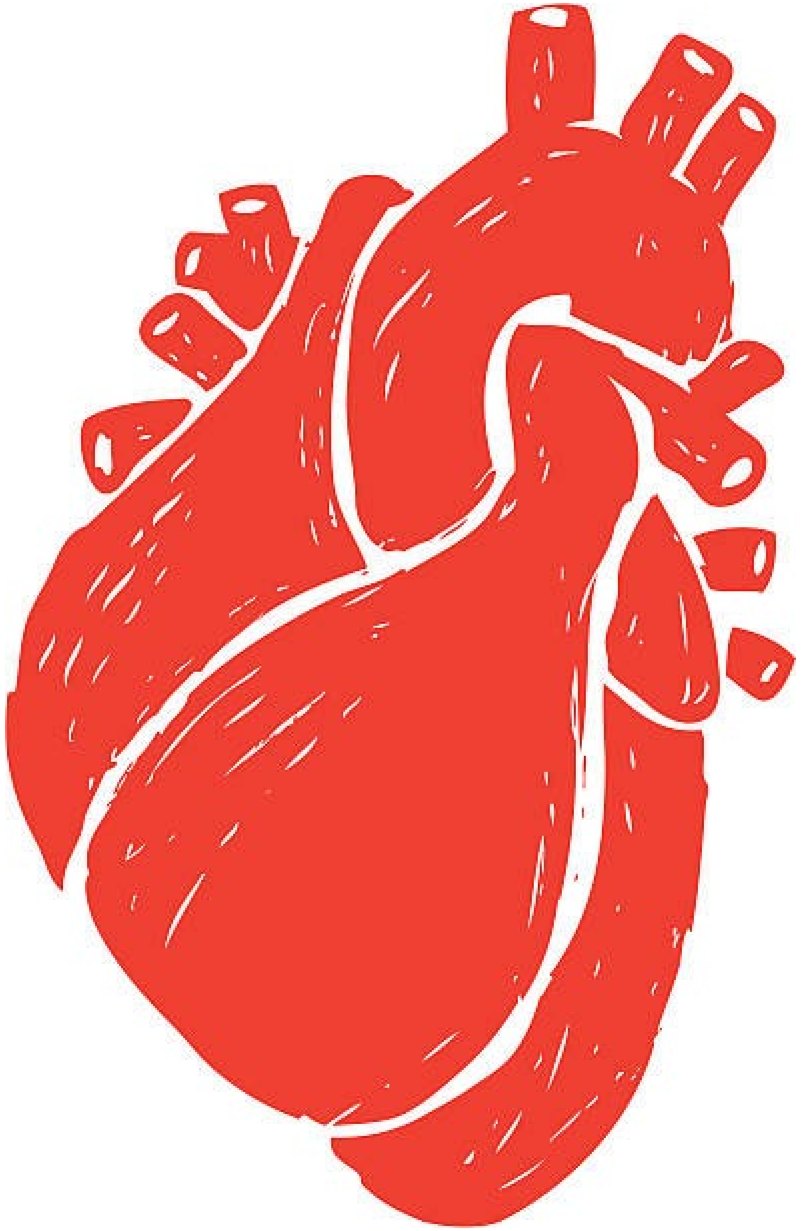

Single gene (monogenic) vs Multi gene (polygenic)

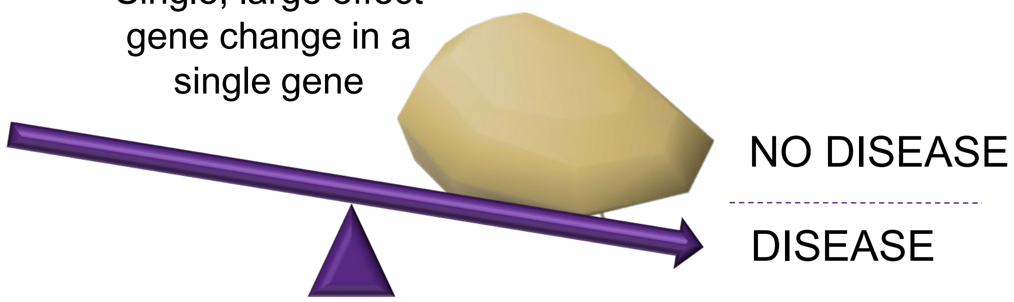

- **Rare**
- **Usually inherited from parent(s)**
- **Smaller impact of environment**

Polygenic risk

- Poly = many
- Genic = genes
- Contribution of multiple genetic changes
- Can estimate genetic risk

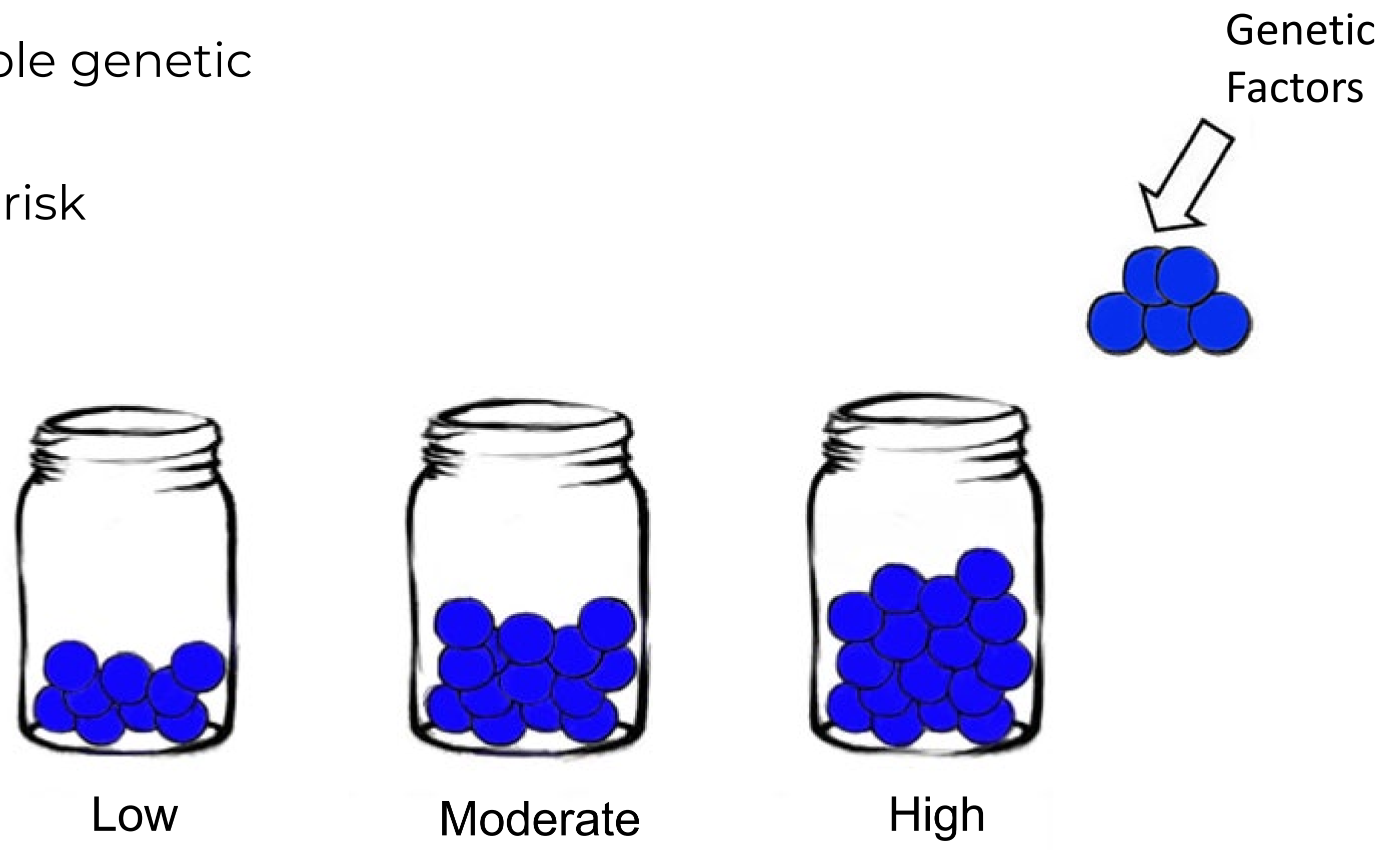

Polygenic risk

- Poly = many
- Genic = genes
- Contribution of multiple genetic changes
- Can estimate genetic risk but…
  - Does not account for non-genetic factors
  - Can combine genetic and non-genetic risk factors

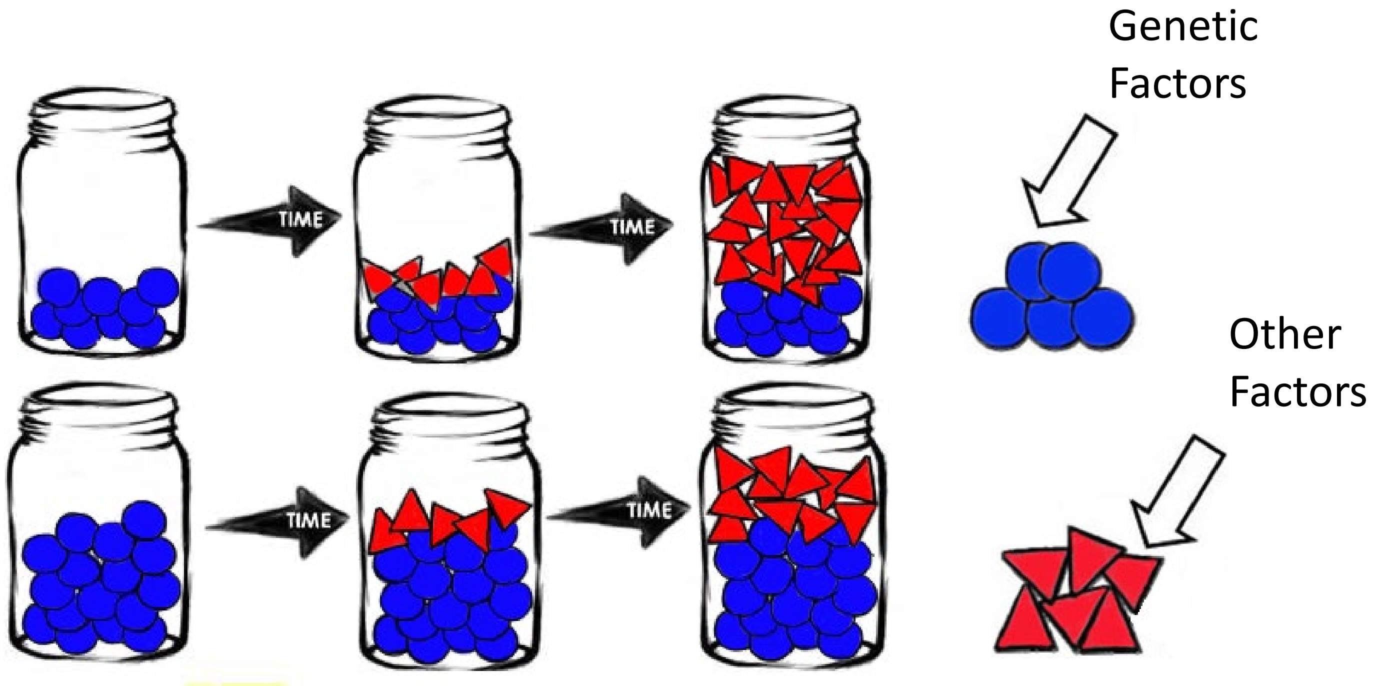

Polygenic risk

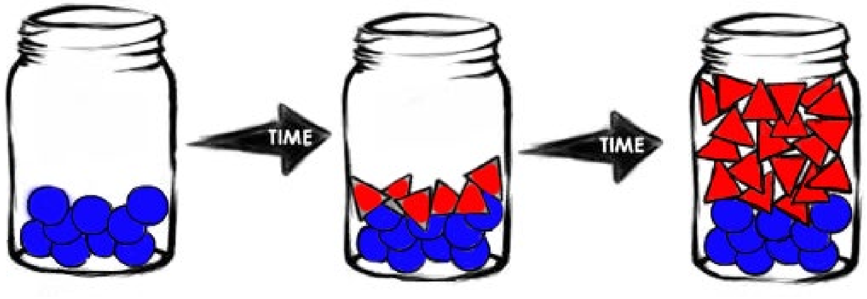

Genetic Factors

Other Factors

Non-genetic factors

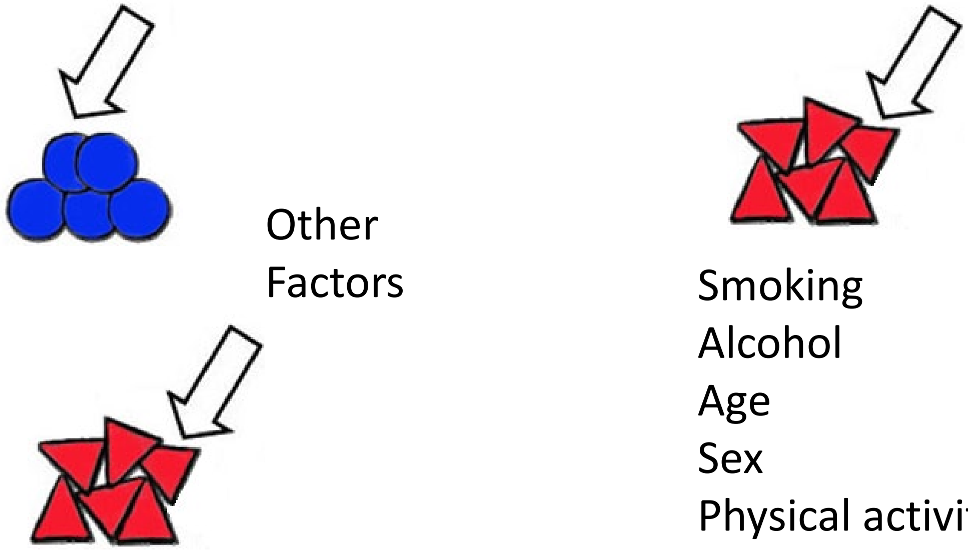

Smoking
Alcohol
Age
Sex
Physical activity
Diet
Stress
Environment
…

Polygenic diseases and traits

Alzheimer's disease

eye colour

asthma

arthritis

height

cancer

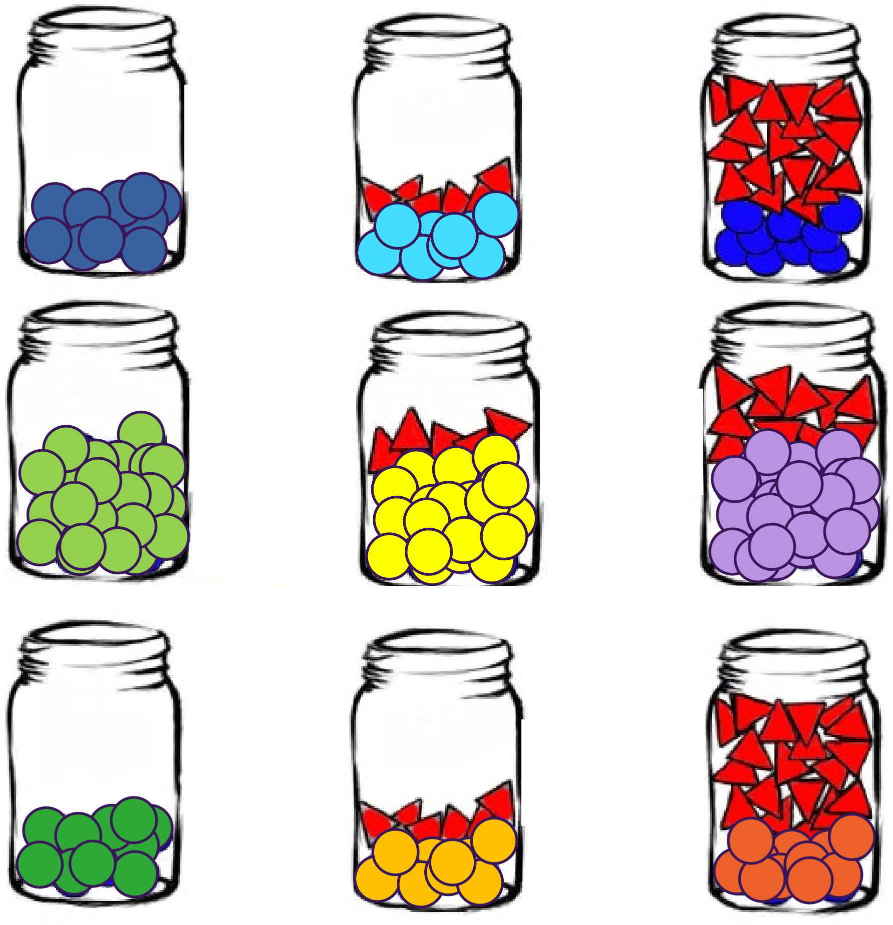

Heart disease

skin colour

schizophrenia

Type 2 diabetes

autism

Monogenic vs Polygenic

Genetic cause of disease

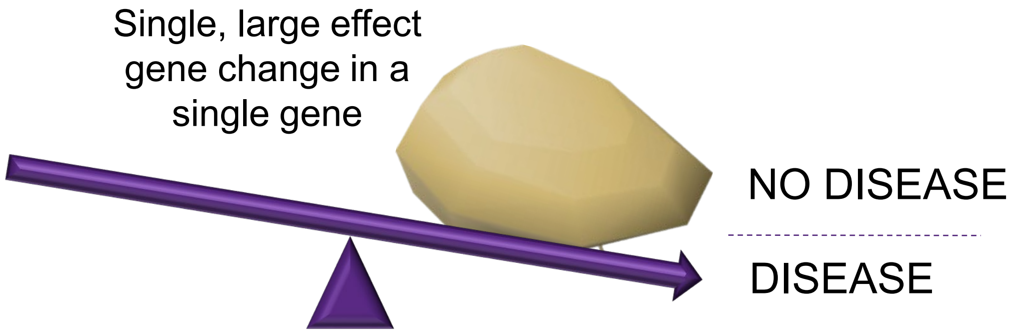

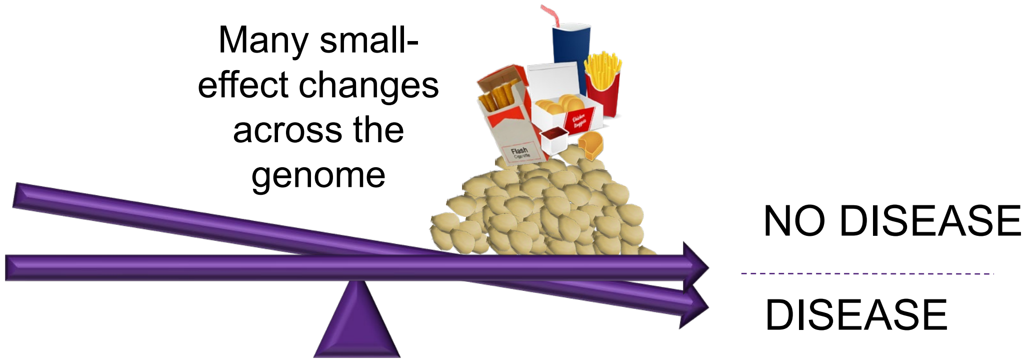

Polygenic risk and heart disease

- Using the many common genetic changes we carry to predict risk
- Know which genetic changes based on population-scale data
- Risk score to personalise health care

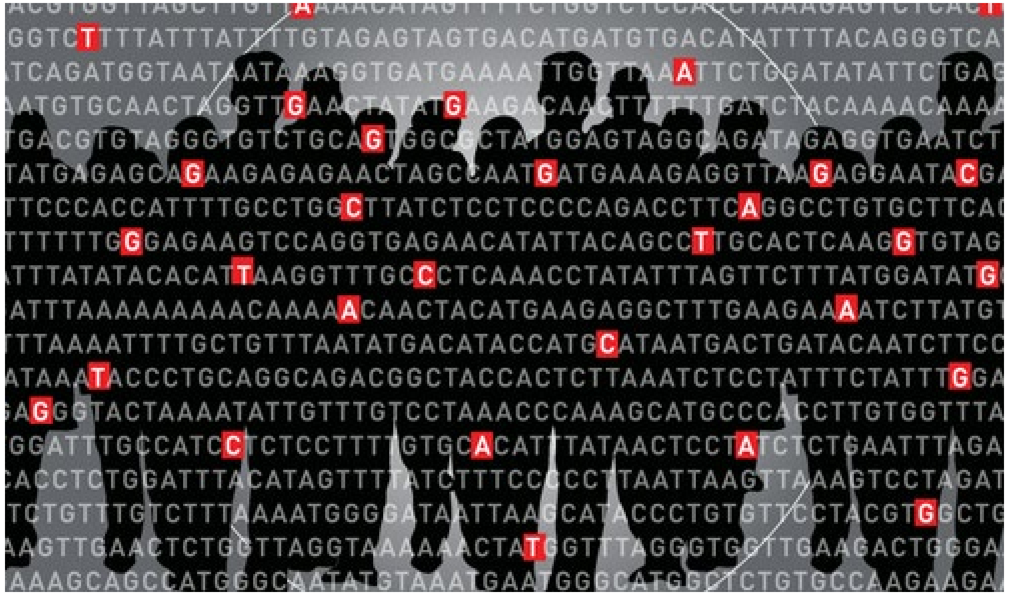

Importance of ancestry

- South Asians make up around **23**% of the world's population and approximately **7**% of Australia's population but…

- Only **1.3**% of genetic study participants are South Asian

- Around 86% of genetic databases are based on European people

- More accurate polygenic risk for people of European ancestry

- Impacts use in healthcare

- Similar lack of diverse data across healthcare

- Further exacerbates disparity

Question

6. How does this information fit with your understanding of risk of heart disease?

Question

7. What do you think would help support our South Asian community be more involved in genetic research for heart disease?

Question

8. What would it mean to you to get a genetic test result about your risk of developing heart disease?

OUTLINE

01

Introductions

02

Genetics presentation

03

Genetics and heart disease

04

Conclusion

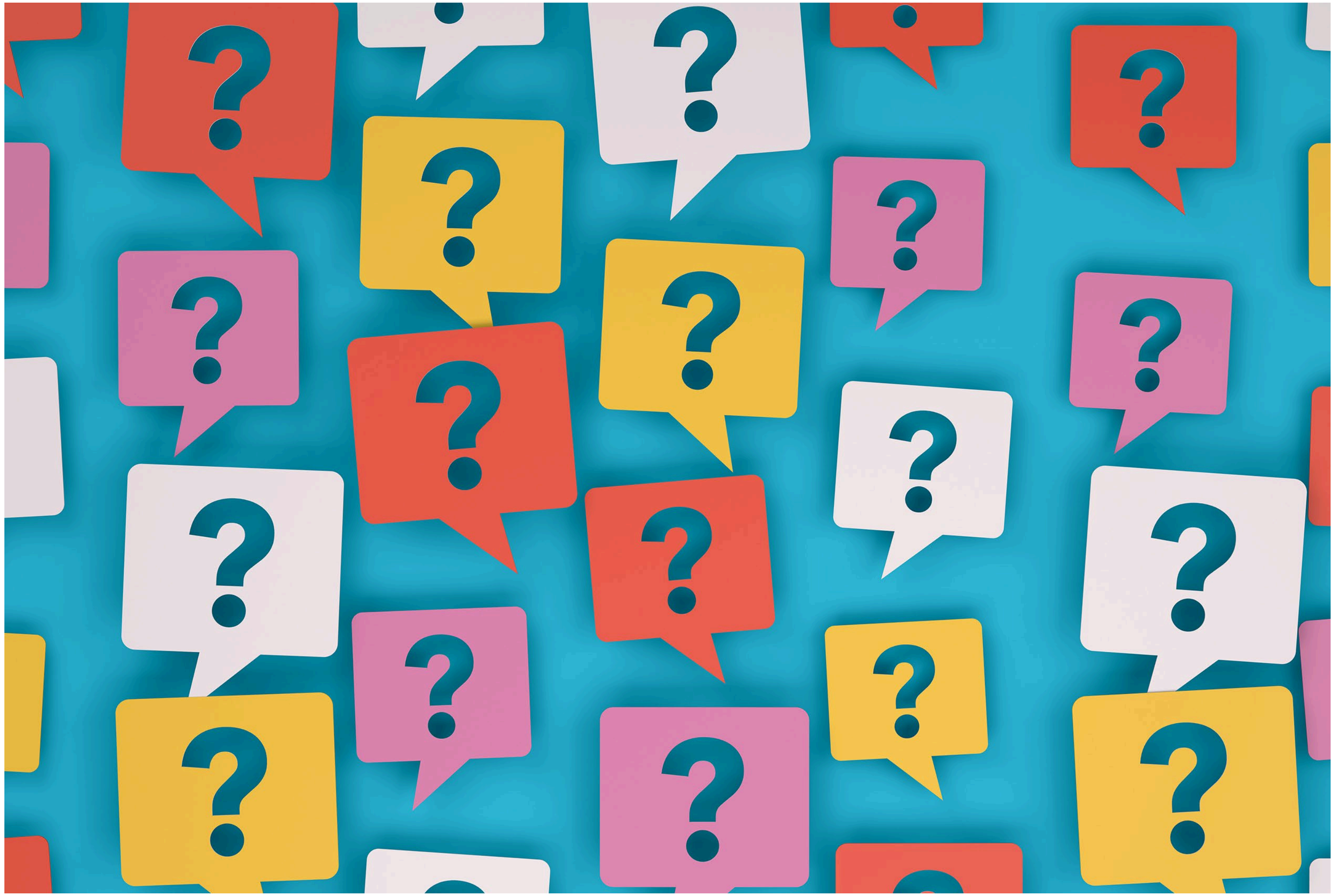

Questions?

Thank you

Contact us:

Phone: +61 7 3443 7499

Email: sagha@uq.edu.au

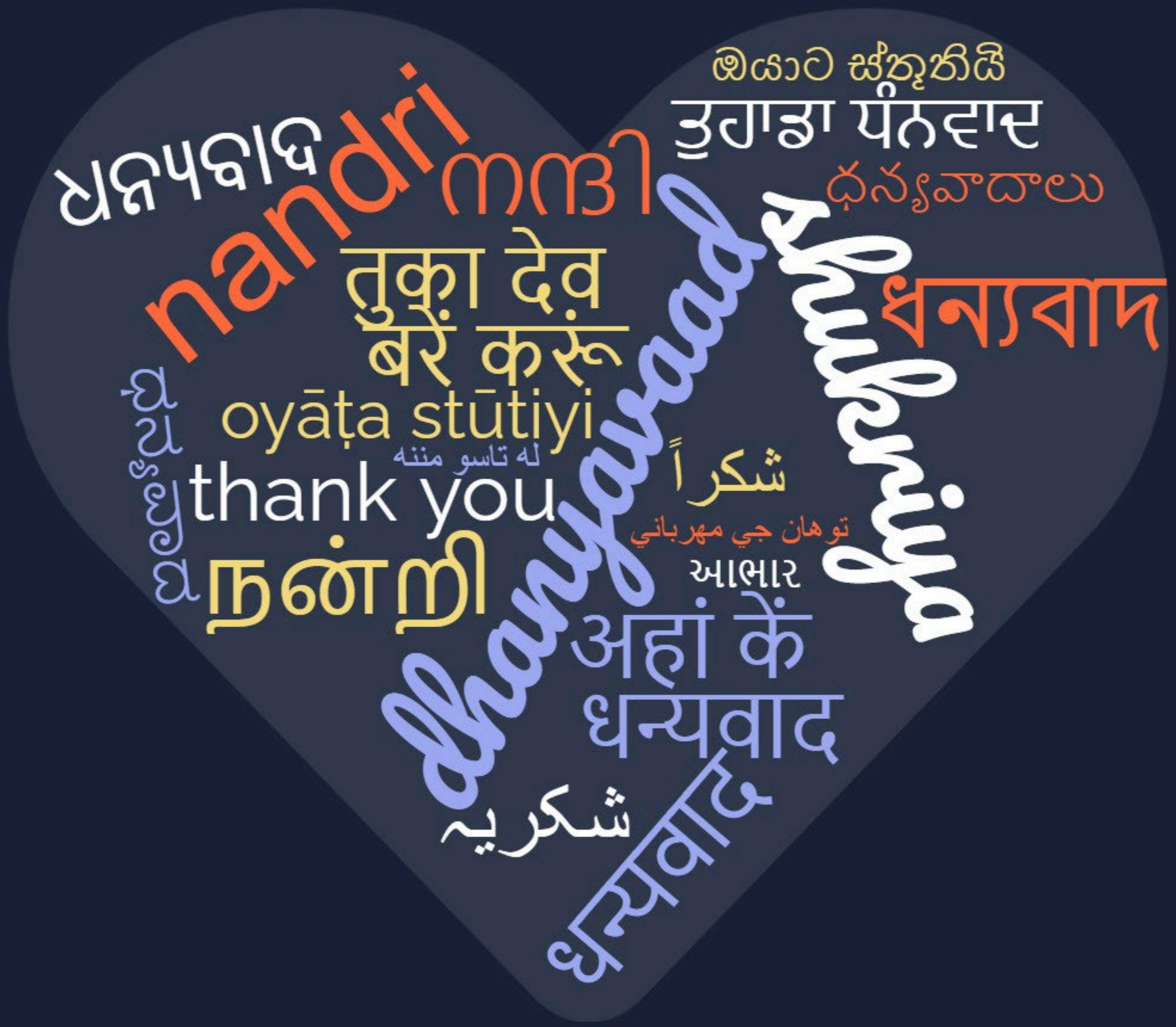

Supplement: Supplementary file 1 — Focus group presentation [file 41431_2026_2129_MOESM1_ESM.pdf]
