# Supplementary material for: “There’s no representation”: a qualitative study of attitudes and motivations towards genomic research participation among Australian South Asians
Source: Eur J Hum Genet. 2026 May 21;34(8):1087–96. doi: 10.1038/s41431-026-02129-3 (PMC13424584; doi:10.1038/s41431-026-02129-3)
Supplement: Supplementary file 2 — Consortia membership [file 41431_2026_2129_MOESM2_ESM.docx]

**Supplementary Materials 2**

**The South Asian Genes and Health in Australia (SAGHA) research team**

Sonia Shah

Tatiane Yanes

Kim Greaves

Julie McGaughran

John Atherton

Katharine Wallis

Rehan Villani

Aideen McInerny-Leo

Divya Mehta

Heena Akbar

Vaishnavi Nathan

Deborah Gilroy

Anjali Henders

Leanne Wallace

Madhura Bhadravathi Lokeshappa
